# Update on 13 Syndromes Affecting Craniofacial and Dental Structures

**DOI:** 10.3389/fphys.2017.01038

**Published:** 2017-12-14

**Authors:** Theodosia N. Bartzela, Carine Carels, Jaap C. Maltha

**Affiliations:** ^1^Department of Orthodontics, Dentofacial Orthopedics and Pedodontics, Charité—Universitätsmedizin, Berlin, Germany; ^2^Department of Orthodontics, Aristotle University of Thessaloniki, Thessaloniki, Greece; ^3^Department of Oral Health Sciences, KU Leuven, Leuven, Belgium; ^4^Department of Orthodontics and Craniofacial Biology, Radboud University Medical Center, Nijmegen, Netherlands

**Keywords:** syndromes, oral manifestations, dental dysmorphologies, craniofacial characteristics, genetics

## Abstract

Care of individuals with syndromes affecting craniofacial and dental structures are mostly treated by an interdisciplinary team from early childhood on. In addition to medical and dental specialists that have a vivid interest in these syndromes and for whom these syndromes are of evident interest, experts of scientific background—like molecular and developmental geneticists, but also computational biologists and bioinformaticians—, become more frequently involved in the refined diagnostic and etiological processes of these patients. Early diagnosis is often crucial for the effective treatment of functional and developmental aspects. However, not all syndromes can be clinically identified early, especially in cases of absence of known family history. Moreover, the treatment of these patients is often complicated because of insufficient medical knowledge, and because of the dental and craniofacial developmental variations. The role of the team is crucial for the prevention, proper function, and craniofacial development which is often combined with orthognathic surgery. Although the existing literature does not provide considerable insight into this topic, this descriptive review aims to provide tools for the interdisciplinary team by giving an update on the genetics and general features, and the oral and craniofacial manifestations for early diagnosis. Clinical phenotyping together with genetic data and pathway information will ultimately pave the way for preventive strategies and therapeutic options in the future. This will improve the prognosis for better functional and aesthetic outcome for these patients and lead to a better quality of life, not only for the patients themselves but also for their families. The aim of this review is to promote interdisciplinary interaction and mutual understanding among all specialists involved in the diagnosis and therapeutic guidance of patients with these syndromal conditions in order to provide optimal personalized care in an integrated approach.

## Introduction

There is a wide spectrum of syndromes that include dental, oral, and craniofacial abnormalities. The range of these disorders encompasses over 1/3 of all congenital malformations (Twigg and Wilkie, 2015). Ideally, interdisciplinary teams in which medical and dental specialists collaborate are treating patients with these syndromes. Medical specialists like pediatricians and geneticists focus on general health issues related to diagnosis and prognosis of the condition, having a holistic view of the patient, while maxillofacial surgeons deal with (major) facial corrections. Dental specialists like pediatric dentists and orthodontists primarily work with caries prevention, diagnosis, and treatment of structural tooth abnormalities, tooth size-shape discrepancies, deviations in tooth number and treatment of malocclusions or facial growth disturbances.

Contrary to the general and genetic diagnosis, most dental abnormalities can only be identified after the first years of life. This delays the dental and orofacial components of the syndromic diagnosis, which are, however, vital for the evaluation of prognostic factors and for the proper timing and management of oral function and, aesthetics as well as for social aspects. On the other hand, early genetic testing, leading to a molecular genetic diagnosis, can be crucial to establish an optimal (therapeutic) strategy, with the ultimate goal to improve the quality of life for these patients.

In addition to the traditional specialists, specialists with a more basic research profile in molecular life sciences become more frequently involved in refined genomics diagnostic processes; moreover the therapeutic planning is expected to further evolve toward the direction of precision medicine aided by pharmacogenomics approaches, with familial transgenerational counseling and personalized preventive strategies ahead. Here, we therefore also provide an update on the advances in the genetic etiology, in genotype-phenotype relations and eventual therapeutic strategies for 13 selected rare syndromes with emphasis on the associated dental and orofacial features. Specifically for the advances in genetic etiology, this update also aims to summarize the Super-Pathways where the causal genetic products operate, as well as their terms for Human Phenotype Ontology (HPO) and Gene Ontology (GO) on locations, molecular functions, and biological processes involved.

Overall, our primary aim with this review is to promote interdisciplinary interaction and mutual understanding among all specialties involved in the diagnosis and therapeutic guidance of patients presenting these syndromes, in order to provide personalized care in an integrated approach.

## Method

For this descriptive review, we selected 13 syndromes based on a combination of criteria:

1. Supportive evidence (in literature or online databases; e.g., OMIM) for presence of associated facial, oral, and/or dental conditions.
2. Prevalence of the disorders around 1/100,000 of the population or higher.
3. Evidence on genomic locus/loci association or causal gene.

Furthermore, we excluded syndromes with a predominant cranial component (e.g., like craniosynostosis syndromes), or syndromal conditions on which a review was recently published (like orofacial clefts with tooth agenesis; Phan et al., 2016).

We adopted the following categories of syndromes involving gingivodental tissues (I), branchial arches (II), orofacial clefts (III), and unusual faces (IV).

In each section, we provide a paragraph with genetics and general features (including potential etiological mechanisms, and potential novel therapeutic considerations), craniofacial features, and oral and dental features. Furthermore, we summarize highly ranked molecular pathways in which the respective causal gene products are reported to be active, as well as the Human Phenotype Ontology (HPO) terms for each of the syndromes/conditions (Table 1A). Moreover, Gene Ontology (GO) terms for localization, molecular function, and biological functions of the genes known to underlie the 13 selected orofacial syndromes, are listed in Table 1B. Additional information for each of the conditions is available in Table S1 and includes the names and aliases of the genes and disorders, their MIM and ORPHA numbers, the type of inheritance and the estimated prevalence. Table 2 provides syndromes that did not fulfill the three criteria to be included in this review.

**Table 1A:** Genes, Genomic Locations, prioritized (Super) Pathways, and Human Phenotype Ontology (HPO) terms (original citation: current source: http://pathcards.genecards.org/Search/Results?query=gene) for each of the 13 selected syndromes (with 21 entities).

**Category/Syndrome**	**Chrom locus**	**Gene(s)**	**SuperPathways**	**Human Phenotype Ontology (HPO) terms**
**I. SYNDROME INVOLVING GINGIVODENTAL TISSUES**				
Neurofibromatosis	17q11	*NF1*	Signaling by PTK6 G-protein signaling M-RAS regulation pathway Syndecan-2-mediated signaling events (3 of 25 superpathways)	HP:0009023 Abdominal wall muscle weakness HP:0001626 Abn. of the cardiovascular system HP:0011039 Abn. of the helix HP:0100763 Abn. of the lymphatic system HP:0000765 Abn. of the thorax
**II. SYNDROMES INVOLVING BRANCHIAL ARCHES**				
Hemifacial microsomia	14q32	*OTX2*	Embryonic and Induced Pluripotent Stem Cell Differentiation Pathways and Lineage-specific Markers Dopaminergic Neurogenesis Mesodermal Commitment Pathway TP53 Network	HP:0040086 Abn. prolactin level HP:0030680 Abn. of cardiovascular system morphology HP:0009888 Abn. of secondary sexual hair HP:0001291 Abn. of the cranial nerves HP:0008187 Absence of secondary sex characteristics
	3q29	*ATP13A3*	/	/
	3q29	*XXYLT1*	/	/
	20q13.33	*MYT1*	Cyclins and Cell Cycle Regulation Mitotic Roles of Polo Like Kinases Mitotic G1-G1/S phases	/
	13 assoc loci	*ROBO1, GATA3, GBX2, FGF3, NRP2, EDNRB, SHROOM3, SEMA7A, PLCD3, KLF12 & EPAS1* (important genes in these loci)	Neural crest cell (NCC) development and Vasculogenesis	/
Treacher Collins syndrome (TCS1)	5q32	*TCOF1*	Ribosome biogenesis in eukaryotes (only 1 superpathway)	HP:0004348 Abn. of bone mineral density HP:0030680 Abn. of cardiovascular system morphology HP:0000682 Abn. of dental enamel HP:0006482 Abn. of dental morphology
Treacher Collins syndrome (TCS2)	13q12.2	*POLR1D*	Assembly of RNA Polymerase-I Initiation Complex Inhibition of Ribosome Biogenesis by p14(ARF) RNA Polymerase I Promoter Escape (3 of 14 superpathways)	HP:0004348 Abn. of bone mineral density HP:0000682 Abn. of dental enamel HP:0006482 Abn. of dental morphology HP:0000834 Abn. of the adrenal glands HP:0000925 Abn. of the vertebral column
Treacher Collins syndrome (TCS3)	6p21.1	*POLR1C*	Assembly of RNA Polymerase-I Initiation Complex Inhibition of Ribosome Biogenesis by p14(ARF) RNA Polymerase I Promoter Escape (3 of 13 superpathways)	HP:0004348 Abn. of bone mineral density HP:0000682 Abn. of dental enamel HP:0006482 Abn. of dental morphology HP:0000834 Abn. of the adrenal glands HP:0000356 Abn. of the outer ear
Möbius syndr (MBS1)	13q12.2-q13	/	/	/
MBS2	3q21-q22	/	/	
MBS3	10q21	/	/	/
MBS4	1p22.5	/	/	/
**III. SYNDROMES INVOLVING OROFACIAL CLEFTS**				
Velocardiofacial syndrome (VCFS)	22q11.2 (del syndr)	*TBX1*	FTO Obesity variant mechanism Heart Development Mesodermal Commitment Pathway (3 of 3 superpathways)	HP:0002101 Abn. lung lobation HP:0000682 Abn. of dental enamel HP:0010978 Abn. of immune system physiology HP:0001939 Abn. of metabolism/homeostasis HP:0012303 Abn. of the aortic arch
Ectodermal dysplasia, Ectrodactyly, Cleft syndrome (EEC3)	3q28	*TP63*	Hypothetical craniofacial development pathway TP53 Network TP53 Regulates Transcription of Cell Death Genes (3 of 14 superpathways)	HP:0004691 2-3 toe syndactyly HP:0000682 Abn. of dental enamel HP:0006482 Abn. of dental morphology HP:0004378 Abn. of the anus HP:0000056 Abn. of the clitoris
Kabuki syndrome	12q13	*KMT2D*	Deactivation of the beta-catenin transactivating complex Lysine degradation PKMTs methylate histone lysines (3 of 8 superpathways)	HP:0007477 Abn. Dermatoglyphics HP:0001671 Abn. of the cardiac septa HP:0000164 Abn. of the teeth HP:0003468 Abn. of the vertebrae HP:0002023 Anal atresia
	Xp12	*KDM6A*	Pathways Affected in Adenoid Cystic Carcinoma Chromatin Regulation/Acetylation Transcriptional misregulation in cancer (3 of 7 superpathways)	HP:0007477 Abn dermatoglyphics; HP:0000769 Abn. of the breast; HP:0001671 Abn. of the cardiac septa HP:0000164 Abn. of the teeth
Kallmann syndrome	Xp22.3	*ANOS1 (&PROKR2)*	Negative regulation of FGFR1 signaling Signaling by FGFR2 Signaling by GPCR (3 of 3 superpathways)	HP:0000002 Abn. of body height HP:0030680 Abn. of the cardiovascular system morphology HP: 0000551 Abn. of color vision HP:0000164 Abn. of the teeth
	10q26.13	*FGFR2(&FGF8, &GNRHR)*	Signaling by FGFR2 Signaling by FGFR2 fusions Downstream signaling of activated FGFR2 (3 of 55 superpathways)	HP:0001233 2-3 finger syndactyly HP:0004691 2-3 toe syndactyly HP:0001999 Abn. facial shape HP:0003312 Abn. form of the vertebral bodies HP:0001627 Abn. heart morphology
Pierre Robin sequence	17q24.3	*SOX9*	Deactivation of the beta-catenin transactivating complex Neural Stem Cell Diff. Pathways and Lineage-specific markers Endochondral ossification (3 of 11 superpathways)	HP:0001627 Abn heart HP:0012856 Abn. scrotal rugation HP:0012244 Abn. sex determination HP:0030680 Abn. of cardiovascular system morphology
van der Woude syndrome	1q32.2	*IRF6*	Hypothetical Craniofacial Development Pathway NF-kB (NFkB) Pathway Primary Focal Segmental Glomerulosclerosis FSGS (3 of 11 superpathways)	HP:0001597 Abnormality of the nail HP:0000772 Abnormality of the ribs HP:0010286 Abnormality of the salivary glands
	1p36.11	*GRHL3*	/	HP:0010286 Abnormality of the salivary glands HP:0000674 Anodontia HP:0000175 Cleft palate HP:0000204 Cleft upper lip HP:0009755 Ankyloblepharon
**IV. SYNDROMES INVOLVING UNUSUAL FACES**				
Coffin-Lowry syndrome	Xp22.12	*RSK2/RPS6KA3*	/	HP:0000940 Abn. diaphysis morphology; HP:0003312 Abn. form of the vertebral bodies; HP:0006482 Abn. of dental morphology; HP:0002269 Abn. of neuronal migration; HP:0007703 Abn. of retinal pigmentation
Opitz GBBB syndrome	Xp22.2	*MID1*	Import of palmitoyl-CoA into the mitochondrial matrix Ubiquitin mediated proteolysis Interferon gamma signaling (3 of 7 superpathways)	HP:0001627 Abn. heart morphology HP:0001739 Abn. of the nasopharynx HP:0001274 Agenesis of corpus callosum HP:0002023 Anal atresia
	22q11.23	*SPECC1L*	/	HP:0011039 Abnormality of the helix HP:0000077 Abnormality of the kidney HP:0000924 Abnormality of the skeletal system HP:0000069 Abnormality of the ureter
Smith-Lemli-Opitz syndrome	11q13.4	*DHCR7*	Cholesterol biosynthesis I Metabolism Terpenoid backbone biosynthesis Vitamin D (4 of 5 superpathways)	HP:0004691 2-3 toe syndactyly HP:0007477 Abnormal dermatoglyphics HP:0003312 Abnormal form of the vertebral bodies HP:0100542 Abnormal localization of kidney HP:0002101 Abnormal lung lobation

*Abn., Abnormal; Abnormality*.

**Table 1B:** Genes, Genomic Locations, and Ontology (GO) terms for location, molecular function, and biological processes (original citation: Ashburner et al., 2000; current source: http://pathcards.genecards.org/Search/Results?query=gene) for the 13 selected syndromes (21 different entities).

**Syndrome/Condition**	**Chromosome locus**	**Gene(s)**	**Gene Ontology (GO) terms for location**	**GO terms or molecular function**	**GO terms for biological process**
**I. SYNDROME INVOLVING GINGIVODENTAL TISSUES**					
Neurofibromatosis type 1	17q11	*NF1*	GO:0005634 nucleus GO:0005730 nucleolus GO:0005737 cytoplasm GO:0005829 cytosol GO:0016020 membrane	GO:0005096 GTPase activator activity GO:0005515 protein binding GO:0008289 lipid binding GO:0008429 phosphatidylethanolamine binding GO:0031210 phosphatidylcholine binding	GO:0000165 MAPK cascade GO:0001649 osteoblast differentiation GO:0001656 metanephros development GO:0001666 response to hypoxia GO:0001889 liver development
**II. SYNDROMES INVOLVING BRANCHIAL ARCHES**					
Hemifacial microsomia	14q32	*OTX2*	GO:0005634 nucleus GO:0030426 growth cone GO:0043234 protein complex	GO:0000978 RNA pol II core promoter proximal region seq-specific DNA binding GO:0001077 transcript activator activity, RNA pol II core promoter proximal region seq-specific binding GO:0003677 DNA binding GO:0003700 transcription factor activity, sequence-specific DNA binding GO:0005515 protein binding	GO:0006355 regulation of transcription, DNA-templated GO:0006366 transcription from RNA polymerase II promoter GO:0006461 protein complex assembly GO:0007275 multicellular organism development GO:0007411 axon guidance
	3q29	*ATP13A3*	GO:0005887 integral component of plasma membrane GO:0016020 membrane GO:0016021 integral component of membrane GO:0043231 intracellular membrane-bounded organelle	GO:0005388 calcium-transporting ATPase activity GO:0005524 ATP binding GO:0016787 hydrolase activity GO:0016887 ATPase activity GO:0046872 metal ion binding	GO:0006812 cation transport GO:0006874 cellular calcium ion homeostasis GO:0070588 calcium ion transmembrane transport GO:0099132 ATP hydrolysis coupled cation transmembrane transport
	3q29	*XXYLT1*	GO:0005783 endoplasmic reticulum GO:0005789 endoplasmic reticulum membrane GO:0016020 membrane GO:0016021 integral component of membrane GO:0030176 integral comp of endoplasmic reticulum membrane	GO:0000287 magnesium ion binding GO:0016740 transferase activity GO:0016757 transferase activity, transferring glycosyl groups GO:0030145 manganese ion binding GO:0035252 UDP-xylosyltransferase activity	GO:0016266 O-glycan processing
	20q13.33	*MYT1*	GO:0005634 nucleus GO:0005654 nucleoplasm GO:0005829 cytosol	GO:0003677 DNA binding GO:0003700 transcription factor activity, sequence-specific DNA binding GO:0008270 zinc ion binding GO:0046872 metal ion binding	GO:0006351 transcription, DNA-templated GO:0006355 regulation of transcription, DNA-templated GO:0007275 multicellular organism development GO:0007399 nervous system development GO:0030154 cell differentiation
Treacher Collins syndrome (TCS1)	5q32	*TCOF1*	GO:0001650 fibrillar center GO:0005634 nucleus GO:0005730 nucleolus GO:0005829 cytosol	GO:0001042 RNA polymerase I core binding GO:0003723 RNA binding GO:0005215 transporter activity GO:0005515 protein binding GO:0046982 protein heterodimerization activity	GO:0001501 skeletal system development GO:0006417 regulation of translation GO:0006810 transport GO:0014029 neural crest formation GO:0014032 neural crest cell development
Treacher Collins syndrome (TCS2)	13q12.2	*POLR1D*	GO:0005634 nucleus GO:0005654 nucleoplasm GO:0005666 DNA-directed RNA polymerase III complex GO:0005736 DNA-directed RNA polymerase I complex GO:0005829 cytosol	GO:0001054 contributes to RNA polymerase I activity GO:0001056 contributes to RNA polymerase III activity GO:0003677 DNA binding GO:0003899 DNA-directed 5-3 RNA polymerase activity GO:0005515 protein binding	GO:0006351 transcription, DNA-templated GO:0006361 transcription initiation from RNA pol I promoter GO:0006362 transcript elongation from RNA pol I promoter GO:0006363 termination of RNA pol I transcription GO:0006383 transcription from RNA pol III promoter
Treacher Collins syndrome (TCS3)	6p21.1	*POLR1C*	GO:0005634 nucleus GO:0005654 nucleoplasm GO:0005666 DNA-directed RNA polymerase III complex GO:0005736 DNA-directed RNA polymerase I complex GO:0005829 cytosol	GO:0001054 contributes to RNA polymerase I activity GO:0001056 contributes to RNA polymerase III activity GO:0003677 DNA binding GO:0003899 DNA-directed 5-3 RNA polymerase activity GO:0005515 protein binding	GO:0006351 transcription, DNA-templated GO:0006361 transcription initiation from RNA pol I promoter GO:0006362 transcript elongation from RNA pol I prom GO:0006363 termination of RNA pol I transcription GO:0006383 transcription from RNA pol III promoter
Möbius syndrome (MBS1)	13q12.2-q13	/	/	/	/
MBS2	3q21-q22	/	/	/	/
MBS3	10q21	/	/	/	/
MBS4	1p22.5	/	/	/	/
**III. SYNDROMES INVOLVING OROFACIAL CLEFTS**					
Velocardiofacial syndrome (VCFS)	22q11.2DS (deletion syndrome)	*TBX1*	GO:0005634 nucleus	GO:0003677 DNA binding GO:0003700 NOT transcription factor activity, sequence-specific DNA binding GO:0042803 protein homodimerization activity GO:0043565 sequence-specific DNA binding	GO:0001525 angiogenesis GO:0001568 blood vessel development GO:0001708 cell fate specification GO:0001755 neural crest cell migration GO:0001934 positive regulation of protein phosphorylation
Ectodermal dysplasia, Ectrodactyly, Cleft syndrome (EEC3)	3q28	*TP63*	GO:0000790 nuclear chromatin GO:0005634 nucleus GO:0005654 nucleoplasm GO:0005667 transcription factor complex GO:0005829 cytoplasm	GO:0000989 transcription factor activity GO:0001077 transcriptional activator activity GO:0002039 p53 binding GO:0003677 DNA binding GO:0003682 chromatin binding	GO:0000122 negative regulation of transcription from RNA pol II promoter GO:0001302 replicative cell aging GO:0001501 skeletal system development GO:0001736 establishment of planar polarity GO:0001738 morphogenesis of a polarized epithelium
Kabuki syndrome	12q13	*KMT2D*	GO:0005634 nucleus GO:0005654 nucleoplasm GO:0035097 histone methyltransferase complex GO:0044666 MLL3/4 complex	GO:0003677 DNA binding GO:0005515 protein binding GO:0008168 methyltransferase activity GO:0008270 zinc ion binding GO:0016740 transferase activity	GO:0001555 oocyte growth GO:0006342 chromatin silencing GO:0006351 transcription, DNA-templated GO:0006355 regulation of transcription, DNA-templated GO:0008284 positive regulation of cell proliferation
	10q26.13	*FGFR2 (&FGF8, &GNRHR)*	GO:0005576 extracellular region GO:0005634 nucleus GO:0005654 nucleoplasm GO:0005737 cytoplasm GO:0005794 Golgi apparatus	GO:0000166 nucleotide binding GO:0004672 protein kinase activity GO:0004713 protein tyrosine kinase activity GO:0004714 transmembrane receptor protein tyrosine kinase activity GO:0005007 fibroblast growth factor-activated receptor activity	GO:0000122 negative regulation of transcription from RNA pol II promoter GO:0000165 MAPK cascade GO:0001525 angiogenesis GO:0001657 ureteric bud development GO:0001701 *in utero* embryonic development
Pierre Robin sequence	17q24.3	*SOX9*	GO:0005634 nucleus GO:0005654 nucleoplasm GO:0005667 transcription factor complex GO:0043234 protein complex GO:0044798 nuclear transcription factor complex	GO:0000976 transcription regulatory region sequence-specific DNA binding GO:0000981 RNA pol II transcription factor activity, sequence-specific DNA binding GO:0001046 core promoter sequence-specific DNA binding GO:0001077 transcriptional activator activity, RNA pol II core promoter proximal region seq-specific binding GO:0001158 enhancer sequence-specific DNA binding	GO:0001501 skeletal system development GO:0001502 cartilage condensation GO:0001503 ossification GO:0001658 branching involved in ureteric bud morphogenesis GO:0001708 cell fate specification
van der Woude syndrome	1q32.2	*IRF6*	GO:0005634 nucleus GO:0005737 cytoplasm GO:0005829 cytosol GO:0070062 extracellular exosome	GO:0000975 regulatory region DNA binding GO:0003677 DNA binding GO:0003700 transcription factor activity GO:0005515 protein binding	GO:0006351 transcription, DNA-templated GO:0006355 regulation of transcription, DNA-templated GO:0007050 cell cycle arrest GO:0008285 negative regulation of cell proliferation GO:0030154 cell differentiation
	1p36.11	*GRHL3*	GO:0005634 nucleus GO:0005654 nucleoplasm	GO:0001228 transcriptional activator activity, RNA pol II transcription regulatory region seq-specific binding GO:0003677 DNA binding GO:0005515 protein binding GO:0031490 chromatin DNA binding GO:0043565 sequence-specific DNA binding	GO:0001736 establishment of planar polarity GO:0001843 neural tube closure GO:0006351 transcription, DNA-templated GO:0006355 regulation of transcription, DNA-templated GO:0006366 transcription from RNA polymerase II promoter
**IV. SYNDROMES INVOLVING UNUSUAL FACES**					
Coffin-Lowry syndrome	Xp22.12	*RSK2/RPS6KA3*	GO:0005634 nucleus GO:0005654 nucleoplasm GO:0005737 cytoplasm GO:0005829 cytosol	GO:0000287 magnesium ion binding GO:0004672 protein kinase activity GO:0004674 protein serine/threonine kinase activity GO:0005515 protein binding GO:0005524 ATP binding	GO:0001501 skeletal system development GO:0002224 toll-like receptor signaling pathway GO:0006468 protein phosphorylation GO:0006915 apoptotic process GO:0007049 cell cycle
Opitz GBBB syndrome	Xp22.2	*MID1*	GO:0005622 intracellular GO:0005737 cytoplasm GO:0005819 spindle GO:0005829 cytosol GO:0005856 cytoskeleton	GO:0005515 protein binding GO:0008017 microtubule binding GO:0008270 zinc ion binding GO:0016740 transferase activity GO:0031625 ubiquitin protein ligase binding	GO:0000226 microtubule cytoskeleton organization GO:0007026 negative regulation of microtubule depolymerization GO:0032874 post regulation of stress-activated MAPK cascade GO:0035372 protein localization to microtubule
	22q11.23	*SPECC1L*	GO:0005815 microtubule organizing center GO:0005819 spindle GO:0005829 cytosol GO:0005856 cytoskeleton GO:0005921 gap junction	/	GO:0007026 negative regulation of microtubule depolymerization GO:0007049 cell cycle GO:0007155 cell adhesion GO:0016477 cell migration GO:0030036 actin cytoskeleton organization
Smith-Lemli-Opitz syndrome	11q13.4	*DHCR7*	GO:0005640 nuclear outer membrane GO:0005783 endoplasmic reticulum GO:0005789 endoplasmic reticulum membrane GO:0005829 cytosol	GO:0009918 sterol delta7 reductase activity GO:0016491 oxidoreductase activity GO:0016628 oxidoreductase activity, acting on the CH-CH group of donors, NAD, or NADP as acceptor GO:0047598 7-dehydrocholesterol reductase activity GO:0050661 NADP binding	GO:0001568 blood vessel development GO:0006629 lipid metabolic process GO:0006694 steroid biosynthetic process GO:0006695 cholesterol biosynthetic process GO:0008202 steroid metabolic process

**Table 2:** Syndromes affecting craniofacial and dental structures below cut off, as not meeting the three inclusion criteria of this review.

**Syndrome**	**Prevalence/100,000**	**OMIM ID**	**Orpha ID**
Cornelia de Lange	1–9	#300590, 610759, 614701, 300882, 122470	199
Apert	1–9	#101200	87
Crouzon	0.1–0.9	#123500, 612247, 101200	207
Down	10–50	#190685	870
Fetal alcohol	Unknown	–	1,915
Holoprosencephaly (non-syndromic)	Unknown	#142945, 142946, 147250, 157170, 236100, 605934, 609408, 609637, 610828, 610829, 612530, 614226	2,162
Marfan	10–50	#616914, 154700	558
Silver-Russell	0.1–0.9	#180860, 312789, 616489	
Smith-Magenis	1–9	#182290	819
Sotos	1–9	#617169, 117550	821
Stickler	1–9	#614134, 614284, 184840, 609508, 604841,108300	828
Turner	10–50 (F)	–	881
Williams	Unknown	#194050	904

*Prevalence, OMIM ID, Orpha ID are provided. F, females*.

## Results

### Syndromes involving gingivodental tissues

Gingivodental syndromes are characterized by gingival hyperplasia and characteristic dental manifestations.

#### Neurofibromatosis type 1

##### Genetics and general features

Neurofibromatosis type 1 (NF1) is a tumor predisposing syndrome which is clinically heterogeneous. It is caused by heterozygous mutations in the neurofibromin gene (*NF1*) on chromosome 17q11 (OMIM # 162200). The incidence is 1 in 2,000–3,000 live births (Uusitalo et al., 2015) and until recently only two genotype-phenotype (G-P) correlations had been identified. The first G-P correlation is the association of *NF1* missense mutations with spinal NF1 and the second one is an association between missense mutations in *NF1* affecting codon p.Arg1809 without externally visible plexiform neurofibromas or cutaneous neurofibromas (Pinna et al., 2015). Neurofibromas are benign tumors which may also occur along nerves and in the proximity of the spinal cord, and in some cases cancerous tumors may develop (Garcia-Romero et al., 2015). In a recent review, a third genotype-phenotype correlation was identified (Kehrer-Sawatzki et al., 2017). This study shows that the recurrent mutations of *NF1* are (micro) deletions comprising the *NF1* gene (with its 57 constitutive and 3 alternatively spliced exons) and its flanking regions. The majority of these deletions encompass 1.4-Mb associated with the loss of 14 protein-coding genes and four microRNA genes, and are correlated with the most severe phenotype of the NF1 spectrum. This not only includes its hallmark features—i.e., café-au-lait spots, iris Lisch nodules, and multiple neurofibromas that are mainly l on or just underneath the skin—but also facial dysmorphologies (see Table S1 in Kehrer-Sawatzki et al., 2017). The clinical features are highly variable and may include hypertension, and skeletal abnormalities such as scoliosis (Ruggieri and Huson, 2001; Garcia-Romero et al., 2015).

##### Craniofacial features

Patients affected with NF1 commonly show macrocephaly, a short mandible, maxilla, cranial base, and low face height (Heerva et al., 2011; Cung et al., 2015). In approximately 20% of the patients, an enlargement of the mandibular canal has been described (Visnapuu et al., 2012).

The prevalence of Class III molar relationship is increased and in sagittal aspect, a marked antegonial notch can be observed in the posterior border of the mandibular ramus, as well as an increased length of the coronoid process and hypoplastic condyles and zygomatic processes (Remberger et al., 1985; Scarano et al., 2005; Bardellini et al., 2011; Javed et al., 2014). Comparing the facial dysmorphology of a NF1 reference group with the patients showing the most severe NF1 phenotype (i.e., with type-1 *NF1* deletions; see previous paragraph), clearly lower frequencies of facial dysmorphology (like asymmetries and hypertelorisms) were observed in the whole NF1 reference group (6–8%) vs. the *NF1* deletions group (28%; see Table 1 in Kehrer-Sawatzki et al., 2017).

##### Oral and dental features

Intra-orally, patients with NF1 usually show unilateral swelling of the gingiva. This may manifest with diffuse enlargements of the attached, and—in some cases—the interproximal gingiva (Javed et al., 2014). These swellings are caused by the plexiform neurofibromas consisting of hypertrophic nerves (Doufexi et al., 2005; Mahajan et al., 2010). Neurofibromas may also be present in other regions of the oral cavity (Sigillo et al., 2002; Scarano et al., 2005). In rare cases, melanin pigmentation of the gingiva is seen.

The timing of the dental development is not different from controls (Jaasaari et al., 2012), but the dental phenotype often comprises impacted, supernumerary, missing, or displaced teeth, and in some cases periapical cementum dysplasia is apparent (Friedrich et al., 2012; Visnapuu et al., 2012). Although the size of the tooth crowns is normal, spacing is often observed (Friedrich et al., 2012).

### Syndromes involving branchial arches

Branchial arch syndromes affect the first and second branchial arch derivatives, and therefore lead to craniofacial deformities (Alfi et al., 2014). The most frequent syndromes in this category are: Hemifacial Microsomia (HFM), Treacher-Collins (TCS) (subdivided in 3 types), and Möbius Syndrome (MBS).

#### Hemifacial microsomia

##### Genetics and general features

Hemifacial Microsomia (HFM) is the most frequent craniofacial condition after cleft lip palate (CLP) and affects 1 in 4,000–5,600 live births (Akram et al., 2015). HFM has many aliases, including Goldenhar syndrome, Oculo-auriculo-vertebral (OAV) spectrum (OAVS) or OAV dysplasia, or Facio-auriculo-vertebral (FAV) sequence. Although it can be inherited in an autosomal dominant (AD) way, most cases are sporadic. Familial cases have been reported, but discordances in monozygotic twins are also present (Akram et al., 2015). Recent findings highlight the genetic heterogeneity of OAVS/HFM (Ballesta-Martínez et al., 2013; Beleza-Meireles et al., 2015; Guida et al., 2015; Zhang et al., 2016; Berenguer et al., 2017). Ballesta-Martínez et al. (2013) described a family with autosomal dominant inheritance of OAVS, and detected a 14q23.1 duplication of 1.34 Mb segregating with the phenotype. This region contains the *OTX2* (Orthodenticle Homeobox 2) gene, encoding a member of the bicoid subfamily of homeodomain-containing transcription factors, which is involved in craniofacial, forebrain, and sensory organ (eyes and ears) development. Therefore, the authors suggested *OTX2* was a good candidate gene for OAVS. Zielinski et al. (2014) failed to identify a pathogenic coding point mutation using whole-exome sequencing in the affected members of a big family. When performing a genome-wide survey of segmental variations they revealed a 1.3 Mb duplication at chromosome 14q22.3 in all affected individuals, that was not present in more than 1,000 chromosomes of ethnically matched controls. Consistent with the heterogeneity of the disorder, they did not identify this duplication in seven additional sporadic HFM cases. When signatures of human craniofacial disease networks, mouse expression data, and predictions of dosage sensitivity were analyzed, they suggested *OTX2* as the most likely causal gene (Zielinski et al., 2014). The fact that OTX2 is also known as an oncogenic driver in medulloblastoma, a condition that was diagnosed in the proband of the family during the course of the study. The authors suggested a role for OTX2 dosage sensitivity in human craniofacial development and raised the possibility of a shared etiology between a subtype of HFM and medulloblastoma (Zielinski et al., 2014). Beleza-Meireles et al. (2015) performed deep phenotyping in 51 patients with OAVS and their parents, and comparative genomic hybridization microarrays to identify potential causative loci. In 10 out of 22 index patients screened, 22q11 dosage anomalies were identified in the array-CGH analysis. They suspected that the 22q11 locus (genes or regulatory components) may be associated with symmetric craniofacial appearance, but their findings in OAVS patients suggest that many factors or even not-identified genes rather contribute to the pathogenesis of the syndrome. In 31% of the patients was a family history of OAVS. Ocular, vertebral, heart, neural, renal, brain (associated often with intellectual disability), limb, urogenital, and/or other organ abnormalities were less common (Gorlin et al., 1963; Akram et al., 2015; Beleza-Meireles et al., 2015).

A *de novo* microduplication spanning 723 Kb on chromosome 3q29 was identified to be associated with HFM (Guida et al., 2015); in the microduplicated region 9 genes were mapped, including *ATP13A3* and *XXYLT1* which are respectively known for their role in organogenesis and in Notch pathway regulation. Zhang et al. (2016) identified eight significantly associated loci and 5 suggestive loci with craniofacial microsomia (CFM). These 13 associated loci, harbored by genes like *ROBO1, GATA3, GBX2, FGF3, NRP2, EDNRB, SHROOM3, SEMA7A, PLCD3, KLF12*, and *EPAS1*, were found to be enriched for genes involved in neural crest cell (NCC) development and vasculogenesis. Then whole-genome sequencing was performed on 21 samples from the case cohort, and several novel loss-of-function mutations were identified (Zhang et al., 2016).

Mutations in *MYT1*, the gene encoding myelin transcription factor 1, which is involved in the retinoic acid (RA) pathway, have recently been identified as causal for OAVS (Berenguer et al., 2017). Therefore, other genes in the RA pathways may be good candidates to further elucidate the genetically heterogeneous HFM/OVA syndrome (Berenguer et al., 2017). Apart from genetic factors, early fetal exposure to drugs such as thalidomide, retinoic acid, and primidone implicated in neuro-ectodermal death may lead to a similar phenotype (Gorlin et al., 2001).

##### Craniofacial features

Forty seven out of 51 patients (92%) present with uni- or bilateral (24/23) ear abnormalities that were associated with hearing loss (Beleza-Meireles et al., 2015). HFM was present in 90% of the patients (often associated with facial nerve palsy; FNP). FNP can influence the craniofacial growth asymmetry in addition to the mandibular condyle hypoplasia and the soft tissue discrepancy (Choi et al., 2014). Some of the most common craniofacial features of HFM include hypoplasia of the zygomatic, mandibular and maxillary bones, and facial muscle hypoplasia (Beleza-Meireles et al., 2015). Colobomas of the upper eyelids are common. In some cases, other facial structures, such as the orbit, eye, nose, cranium, or neck, may be involved. Involvement is usually limited to one side, but bilateral involvement is known. Cephalometric analysis shows that patients with HM present an upward cant of the occlusal plane and a smaller mandibular body and ramus at the affected side (Shibazaki-Yorozuya et al., 2014; Brandstetter and Patel, 2016; Heike et al., 2016).

As a consequence, patients with HFM tend to have large and steep gonial angles, a retrognathic mandible, and a mildly convex face in profile (Seow et al., 1998; Ongkosuwito et al., 2013a; Ahiko et al., 2015). Despite their shorter mandibles, mandibular growth rate is similar to the normal population (Ongkosuwito et al., 2013b). CLP is present in ~10% of patients with HFM (Ye et al., 2005). Unilateral Craniofacial Microsomia (UCM) is also reported to be an underappreciated cause of obstructive sleep apnea (OSA). The prevalence of OSA in patients with UCM is up to 10 times higher than in the general population (Szpalski et al., 2015).

##### Oral and dental features

In deciduous and permanent dentition the mesiodistal dimensions of all molars are smaller than in control individuals, supporting the concept that HFM is a bilateral rather than a unilateral condition (Seow et al., 1998). However, the difference is most pronounced in the mandibular permanent first molar on the affected side. The canines and the incisors have normal size, both in the deciduous and in the permanent dentition, although deviating development of the mandibular canines has also been reported (Seow et al., 1998; Ahiko et al., 2015).

The prevalence of tooth agenesis (TA) is higher than in controls, and amounts approximately 25%; the mandibular second premolar and second molars are the ones most affected (Maruko et al., 2001; Ongkosuwito et al., 2010). Compared to non-affected controls, tooth development is delayed in patients with HFM, and only in the most severe cases with an absent mandibular ramus and glenoid fossa, the affected side is significantly more delayed than on the “non-affected” side (Ongkosuwito et al., 2010; Ahiko et al., 2015). About half of the HM children show mild tongue dysmorphology; this feature however seems to be easily overlooked (Chen et al., 2009).

#### Treacher collins

##### Genetics and general features

Treacher Collins Syndrome (TCS), is a largely AD condition with a penetrance of ~90% and variable expressivity (Dixon et al., 2007), affecting 1:50,000 live births. There are mainly 3 types can be discerned with bilateral oto-mandibular dysplasia as a common characteristic in craniofacial development. TCS1 and TCS2 show AD inheritance (suggested with incomplete penetrance), while TCS3 is an AR condition. In most cases the mutation is in the gene *TCOF1* located on 5q32-q33.1 chromosome. TCS1 is caused by mutations in the Treacle Ribosome Biogenesis Factor 1 (*TCOF1*) gene, which encodes a nucleolar phosphoprotein involved in rRNA transcription (Valdez et al., 2004), regulating RNA polymerase I by connecting RNA polymerase I with enzymes responsible for ribosomal processing and modification, essential in normal cell function. It is also required for neural crest specification (Hayano et al., 2003) and is highly expressed in embryogenesis during fusion of the neural tube and in the branchial arches, suggesting a role for the gene in the development of the craniofacial complex (Dixon et al., 1997). TCS2 and TCS3 are caused by mutations in the RNA Polymerase I Subunit D (*POLR1D)* gene at 13q12.2 and RNA Polymerase I Subunit C (*POLR1C*) gene at 6p21.1, respectively. These genes encode RNA polymerases I and III subunits, which are also involved in rRNA transcription (Dauwerse et al., 2011). These findings suggest that TCS is a ribosomopathy (Dauwerse et al., 2011). In 10% of the patients with TCS, the genetic defect for the molecular diagnosis is still unknown. Although so far no clear genotype-phenotype correlation has been documented for TCS, Dixon et al. (2006) showed that Tcof1 haplo insufficiency results in oxidative stress-induced DNA damage with neuroepithelial cell death as a result. Importantly, Sakai et al. (2016) demonstrate that maternal treatment with antioxidants minimizes cell death in the neuroepithelium and substantially ameliorates or even prevents the pathogenesis of craniofacial anomalies in Tcof1(+/−) mice (Sakai et al., 2016). They conclude that antioxidant therapy may provide an avenue of protection against the pathogenesis of TCS and similar neurocristopathies (Sakai et al., 2016).

In a large TCS group of patients, cardiac malformations were present in 12%, while brain, kidney, and limb anomalies were rare (Vincent et al., 2016). In a case report Li et al. (2009) described a patient with TCS who had additional features including an encephalocele, and several extra-craniofacial anomalies involving the thyroid, the thymus, the heart, an accessory spleen, ectopic adrenal gland tissue, and underdeveloped external genitalia (Li et al., 2009).

##### Craniofacial features

Recently, the rate of all clinical features observed in TCS1 were described in 70 patients (Vincent et al., 2016). These authors proved that the vast majority of the symptoms were: craniofacial and comprised downward-slanting palpebral fissures (in 100% of the patients); malar hypoplasia (in 99%); conductive deafness (in 91%); mandibular hypoplasia (in 87%); atresia of external ear canal (in 72%), microtia (in 71%); coloboma of the lower eyelid (in 65%); facial asymmetry (in 53%), and projection of scalp hair onto the lateral cheek (in 48%). Twenty two percent of the patients with TCS1 in this study had a cleft palate and 14% had choanal stenosis or atresia (Vincent et al., 2016).

Moreover, a large proportion of the children with TCS (irrespective the TCS type) are reported to have a narrow arched palate, hypoplasia of the maxilla and a retrognathic mandible (Posnick and Ruiz, 2000). Predominant hypoplasia of soft tissues is observed on the face. Also complex abnormalities in the temporomandibular joint may lead to a limitation of mouth opening (Poswillo, 1975; Posnick and Ruiz, 2000) and an anterior open bite of varying severity (Posnick and Ruiz, 2000; Martelli-Junior et al., 2009; Trainor and Andrews, 2013). While the presence of cleft palate with or without cleft lip is reported in many patients in some studies (Posnick and Ruiz, 2000; da Silva Dalben et al., 2006; Martelli-Junior et al., 2009). Martelli-Junior et al. (2009) reported the absence of orofacial clefts in seven sporadic cases with TCS (Martelli-Junior et al., 2009).

In a prospective case study, which included 19 patients who underwent genetic testing, medical and dental examinations, and polysomnography, disturbed respiration was demonstrated in all participating patients. Eighteen of them met the diagnostic criteria for obstructive sleep apnea syndrome (OSAS) (Akre et al., 2012).

##### Oral and dental features

Dental anomalies have been reported in about 60% of the children with TCS. Tooth agenesis (TA) is the most frequent anomaly and it most commonly affects the mandibular second premolars, followed by maxillary second premolars, lateral incisors, and maxillary canines (da Silva Dalben et al., 2006). Also, impacted maxillary supernumerary teeth, hypoplastic, and malpositioned maxillary central incisors (da Silva Dalben et al., 2006) and ectopic eruption of the maxillary first molars have been reported (da Silva Dalben et al., 2006).

Seven sporadic patients (six females and one male) with TCS without obvious family history for related features, manifested various types of malocclusions, three of which had an anterior open bite and one had a short soft palate (Martelli-Junior et al., 2009).

#### Möbius syndrome

##### Genetics and general features

Möbius syndrome (MBS) is a rare congenital disorder with the preliminary diagnostic criteria of congenital facial and abducent nerve palsy with impairment of ocular abduction. Other cranial nerves are also commonly involved. The prevalence rate of MBS is ~1 in 100,000 live births (Ghosh et al., 2017). The genetic etiology of the syndrome is heterogeneous with four genetic loci described: MBS1 on chromosome 13q12.2-q13, MBS2 on chromosome 3q21-q22, MBS3 on chromosome 10q21 and MBS4 on chromosome 1p22.5. Various other etiologic factors have however been associated with MBS, including vascular interruption in the subclavian artery territory, infections, hyperthermia, trauma, and teratogens such as benzodiazepines, thalidomide, alcohol, cocaine, misoprostol, and ergotamine (Ghosh et al., 2017). Its proximal cause is the abnormal development—or absence—of the 7^th^ cranial nerve (facial) in 100% of patients and of the 6^th^ cranial nerve (abducens) in 75% of them. Occasionally, other cranial nerves can also be affected (Verzijl et al., 2003); moreover, mild intellectual disability can occur in 10% of cases (Verzijl et al., 2003). MBS also comprises a large phenotype variation including variable features such as limb, and musculoskeletal, behavioral, and cognitive abnormalities (Van Der Zwaag et al., 2002; Verzijl et al., 2003; Ghosh et al., 2017). Recently, McClure et al. (2016) reported that MBS is accompanied by an increased rate of several orthopedic problems, including clubfoot, scoliosis, and upper extremity differences that often require surgical treatment. Therefore, early involvement of orthopedic surgeons in the care of patients with MBS is often necessary. Congenital heart diseases and other syndromes are often associated with MBS (Sharma et al., 2015; Budic et al., 2016; Gaudin et al., 2016).

##### Craniofacial features

The first manifestation is sucking impairment and excessive drooling often accompanied by respiratory difficulties (Verzijl et al., 2003). As the child grows, the inability to move the facial muscles and the eyes becomes obvious (Zuker et al., 2000; Sjogreen et al., 2001, 2011; Bianchi et al., 2010). Other anomalies are also commonly associated, such as orofacial dysmorphology and jaw abnormalities (Van Der Zwaag et al., 2002; Verzijl et al., 2003). Children with MBS are commonly born with persisting micrognathia and microstomia (De Serpa Pinto et al., 2002; Magalhaes et al., 2006; Bianchi et al., 2013). The maxilla is mostly narrow and high arched, and cleft palate is present in about 30% of the cases (De Serpa Pinto et al., 2002; Stromland et al., 2002; Magalhaes et al., 2006). In their report on two patients with MBS Ghosh et al. (2017) show a marked facial asymmetry in one of their patients; this patient also suffers from torticollis (Ghosh et al., 2017).

##### Oral and dental features

The time of eruption of the deciduous dentition varies. The upper lip often is hypoplastic, and a frontal open bite is seen in about 50% of the patients (De Serpa Pinto et al., 2002; Magalhaes et al., 2006#1655). Agenesis of the lower second premolars, enamel hypoplasia and crowding has been reported in 30–40% of the cases (Stromland et al., 2002). The presence of a short or fissured and abnormally shaped tongue has been described (De Serpa Pinto et al., 2002). Incompetent lips and failure to close the mouth were the main complaints in the two patients of a case report; a multi-disciplinary approach was planned to correct these features (Ghosh et al., 2017). Patients with MBS have been described with higher risk for caries, most probably due to reduced salivary rate (Castro et al., 2016; Martins Mussi et al., 2016), and higher occurrence of periodontal disease (Martins Mussi et al., 2016).

### Syndromes involving orofacial clefts

Syndromes in this group are all related to the development of cleft lip and/or palate. Apart from that they also include a highly variable spectrum of clinical features, such as ectrodactyly, ectodermal anomalies, musculoskeletal problems, hypogonadism, mental retardation, and hearing loss. The 22q11.2 deletion syndrome (22q11.2DS), is also known as velocardiofacial (VCF), Shprintzen, or DiGeorge syndrome.

#### 22q11.2 deletion syndrome

##### Genetics and general features

The 22q11.2DS has first been described by Shprintzen et al. (1978) as the Velocardiofacial Syndrome (VCFS), and has been extensively studied since then. It is one of the most frequent microdeletion syndromes with an incidence of 2–5 in 10,000 live births and is caused by a 1.5–3.0 Mb hemizygous deletion of chromosome 22q11.2. In particular, haploinsufficiency of the *TBX1* gene is found to be responsible for most of the physical malformations. However, also point mutations in the *TBX1* gene can cause the disorder (Chieffo et al., 1997). Although many cases are sporadic, autosomal dominant inheritance of the disorder has been reported, caused by a 1.5–3.0 Mb hemizygous deletion of chromosome 22q11.2. Haploinsufficiency of the TBX1 gene in particular is accountable for most of the physical malformations. There is evidence that point mutations in the TBX1 gene can also cause the disorder. Typical frequent signs and symptoms originally described by Shprintzen et al. (1981) comprise cleft palate, cardiac anomalies, typical facial characteristics and learning disabilities. Less frequent features in various body parts have been described in many reports on VCFS which may also vary widely among family members. These include dysgenesis of the thymus and parathyroid glands, immune deficiencies, hypocalcaemia, and disturbances in cognitive and behavioral development (Borglum Jensen et al., 1983; Ryan et al., 1997; Wang et al., 1997; Gaspar et al., 1999; Pradel et al., 2003; Yang et al., 2005; Nugent et al., 2010; Toka et al., 2010; Wu et al., 2013). The majority of patients are constitutionally small, including height or weight parameters (Ryan et al., 1997). Because of the high phenotypic variability and the genetic heterogeneity of VCFS, it remains challenging to define its complex genotype-phenotype relation. The description of the animal models moreover guides the reader through the important breakthroughs concerning the etiological mechanisms lying at the origin of VCFS (Stalmans et al., 2003; Liao et al., 2004), and which are directly relevant for clinical diagnosis and will ultimately lead to targeted therapeutic strategies.

##### Craniofacial features

More than 75% of the individuals have cleft palate or palatal anomalies and specific facial characteristics like hypertelorism (Nugent et al., 2010; Toka et al., 2010; Wu et al., 2013). Many patients with VCFS have a short philtrum, thick and reflected lips (Wang et al., 1997; Fukui et al., 2000). The face can appear long and asymmetric, often with hypotonic muscles, and microcephaly (Toka et al., 2010). Typical facial features include a bulbous-tipped nose, malar flattening, narrow alar base, and thin alae nasi. Skeletal alterations include a short velum, a deep cavum, and malformed or short cranial base (Wang S. et al., 2009; Leveau-Geffroy et al., 2011), large cranial base angle (Oberoi and Vargervik, 2005b), micrognathia (Wang et al., 1997), or retrognathia (Gaspar et al., 1999; Wang K. et al., 2009), steep mandibular plane angle, increased anterior face height, retruded chin, retroclined lower incisors, and increased interincisal angle (Oberoi and Vargervik, 2005b; Oberoi et al., 2011). Malocclusions associated with 22q11.2DS are skeletal class II, with a retruded mandible and open bite (Oberoi et al., 2011; Lewyllie et al., 2017). Interestingly, a general trend of facial hypoplasia in the lower part of the face was evidenced with 3D facial analysis in 20 children with 22q11.2DS compared to controls (Lewyllie et al., 2017).

Furthermore, functional impairment can lead to craniospinal growth disorders (Leveau-Geffroy et al., 2011), asymmetric development of the pharynx and larynx, velopharyngeal insufficiency (Nugent et al., 2010; Leveau-Geffroy et al., 2011) and alternations in palatal motion. These developmental abnormalities, increase speech and respiration difficulties (Pradel et al., 2003; Chegar et al., 2006; Kummer et al., 2007).

##### Oral and dental features

Patients with 22q11.2DS show a higher prevalence of missing permanent teeth, especially mandibular incisors, maxillary second premolars, and maxillary lateral incisors (Heliovaara et al., 2011). Despite individual variability, a significantly higher prevalence of tooth agenesis was also observed in 20 children with 22q11.2DS (20%) compared to controls (Lewyllie et al., 2017). A solitary median maxillary or mandibular central incisor has been reported in some cases as well (Oberoi and Vargervik, 2005b; Yang et al., 2005).

The development and eruption of permanent teeth is often delayed, with enamel opacities (Fukui et al., 2000) and hypoplastic alterations (Fukui et al., 2000; da Silva Dalben et al., 2008). Hypomineralizations are observed in both dentitions but in permanent dentition they are twice as frequent as in the primary one (Nordgarden et al., 2012). Impaired salivary flow has also been reported (Toka et al., 2010).

Mitsiadis et al. (2008) showed that the expression of the *Tbx1* gene implicated in human DiGeorge syndrome, requires mesenchyme-derived FGF signaling for tooth development and correlates with determination of the ameloblast lineage. With their long-term culture techniques, allowing unharmed growth of incisors until their full maturity. Caton et al. (2009) showed that incisors of *Tbx1*^−/−^ mice were hypoplastic and lacked enamel. Their further experiments demonstrated that Tbx1 proved essential for the maintenance of ameloblast progenitor cells in rodent incisors and that its deletion results in the absence of enamel formation. These results explain why dental phenotypes like enamel hypoplasia and possibly tooth agenesis, are often found in patients with 22q11.2DS/DiGeorge or VCF syndrome. Using Tbx1 lineage tracing experiments it was shown that *Tbx1* conditional knockout [*Tbx1(cKO)*] mice featured microdontia, coincide with decreased stem cell proliferation (Gao et al., 2015). Their further results also suggested that Tbx1 regulates the proliferation of dental progenitor cells and craniofacial development through miR-96-5p and PITX2. Cleft palate was observed in *Tbx1(cKO)* consistent with the orofacial and tooth defects associated with *TBX1* deletion (Gao et al., 2015).

#### EEC-syndrome

##### Genetics and general features

EEC is an autosomal dominant disorder with variable expression, characterized by the triad of ectrodactyly (“claw-like” hands and feet), ectodermal dysplasia, and orofacial clefts. Celli et al. (1999) mapped EEC3 to 3q27. With mutation analysis of the *TP63* gene the phenotype-genotype relation could be determined for EEC3 with heterozygous mutations in *TP63* (fine mapped on 3q28) as molecular causes of this syndrome. *TP63* encodes multiple isoforms of the p63 transcription factor; its biological role is quite complex, with wide-ranging effects on development and differentiation. As can be observed in *Tp63* knockout mice, the development of stratified epithelia is blocked leading to aplasia of multiple ectodermal appendages (including teeth), as well as orofacial clefting and limb defects (Romano et al., 2012). Ectrodactyly is described in 68–84% and ectodermal dysplasia in 50–77% of the cases (Roelfsema and Cobben, 1996; Rinne et al., 2006).

Ectodermal dysplasia often leads to skin hypopigmentation and dry skin, hyperkeratosis, or atrophy, nail dystrophy, fine and sparse hair and eyebrows, reduced or absence of salivary and sweat glands (Buss et al., 1995; Rinne et al., 2006). Other features in this syndrome are lacrimal tract abnormalities, ophthalmological problems, urogenital abnormalities, mammary gland/nipple hypoplasia, and hearing loss (Maas et al., 1996; Roelfsema and Cobben, 1996; Rinne et al., 2006). Hypothalamopituitary dysfunction (Gershoni-Baruch et al., 1997), growth hormone-deficiency (Knudtzon and Aarskog, 1987), and growth retardation (Roelfsema and Cobben, 1996) have also been described in EEC syndrome.

##### Craniofacial features

Forty to 70% of the patients diagnosed with EEC, have an orofacial cleft (Buss et al., 1995; Roelfsema and Cobben, 1996). One to five percent of the EEC patients exhibit midfacial, zygomatic, maxillary, and mandibular hypoplasia, microcephaly, and premaxillary protrusion. However, these features are not considered typical characteristics of the syndrome (Roelfsema and Cobben, 1996). Interestingly, in Genome-wide meta-analyses of non-syndromic orofacial clefts an association was also found between SNPs located in a *TP63* enhancer and clefts of the lip with or without cleft palate (CL/P) (Leslie et al., 2017).

##### Oral and dental features

EEC patients have primary and permanent dentitions affected (Klein et al., 2013). In some patients where the EEC syndrome was caused by mutations in *TP63*, the primary dentition was normal (Sripathomsawat et al., 2011). *TP63* mutations may have less effect on deciduous dentition but more data is needed to confirm it (Sripathomsawat et al., 2011). However, their permanent dentition is universally affected (Buss et al., 1995; van Bokhoven et al., 2001; Sripathomsawat et al., 2011). The most common dental features are hypodontia (King et al., 1994), or even anodontia (Wallis, 1988), enamel hypoplasia (Leibowitz and Jenkins, 1984; Knudtzon and Aarskog, 1987; Wallis, 1988; King et al., 1994), generalized microdontia (King et al., 1994), or poorly developed (Wallis, 1988) and peg-shaped teeth (Leibowitz and Jenkins, 1984; Wallis, 1988). The dental phenotypes can be explained as *TP63* is expressed in almost all phases of prenatal human tooth development (Kock et al., 2005). In the latter study a positive of *TP63* was observed in both the cap stage and the bell stage in the cells of the oral mucosa, the inner and outer enamel epithelium, and in the primary and secondary dental lamina. In the early cap stage, there is a strong positive *TP63* in the enamel knot, but not in the late cap stage. Therefore, an important regulatory function of *TP63* in the enamel knot was suggested (Kock et al., 2005). Recently, several p63-positive stem cell reservoirs in the non-ameloblast layers of the enamel organ were also evidenced pointing to a role of p63 in stem cell maintenance (Liu et al., 2016). As p63 is important in transcriptional and signaling networks of epithelial cells, its dysregulation is also associated with tumorigenesis (like squamous cell carcinoma), metastasis, and senescence (Nekulova et al., 2011). Moreover, p63 autoantibodies were found to be associated with an increased susceptibility to oral candidiasis (King et al., 1994), and immunologically mediated chronic ulcerative stomatitis (Romano et al., 2012) and with xerostomia and/or deep tongue fissures (King et al., 1994). The dental age and tooth eruption are as well late (Klein et al., 2013).

#### Kabuki syndrome

##### Genetics and general features

Kabuki syndrome (KS) is a congenital mental retardation syndrome with additional features, including postnatal dwarfism and a peculiar facies. It is genetically heterogeneous caused by heterozygous mutation in *MLL2* (now called *KMT2D* gene) on chromosome 12q13, causing Kabuk-1 with autosomal dominant (AD) inheritance, and the *KDM6A* gene on chromosome Xp11.3 causing Kabuki-2 with X-linked dominant inheritance. KS was described for the first time in Japanese children (Kuroki et al., 1981), and has a prevalence of 3.2/100,000 in the Japanese population. It is however more common in non-Japanese populations, especially in patients with orofacial clefting (Burke and Jones, 1995). The syndrome affects many parts of the body. The main characteristics of the syndrome appear later in life, making an early diagnosis difficult. KS patients have developmental delay of variable severity (Adam and Hudgins, 2005; Schrander-Stumpel et al., 2005; Petersen et al., 2010; Cheon and Ko, 2015) and intellectual disability that ranges from mild to severe (Wessels et al., 2002; Schrander-Stumpel et al., 2005; Vaux et al., 2005). Early puberty is frequently reported (Schrander-Stumpel et al., 2005). Skeletal abnormalities such as scoliosis, brachydactyly V, brachymesophalangy, clinodactyly of the fifth fingers, or hypermobility and dislocation of hip and knee joints are often encountered. Affected individuals may also have seizures, or muscle hypotonia, related with a defect of the connective tissue (Burke and Jones, 1995).

Heart abnormalities (Yuan, 2013; Yoon et al., 2015), frequent otitis media and hearing loss (Kawame et al., 1999; Tekin et al., 2006), ocular problems such as nystagmus or strabismus, and ptosis, are often features of the syndrome (Turner et al., 2005).

Van Laarhoven et al. (2015) used morpholino antisense oligonucleotides to knock down Kmt2d in zebrafish, and at 5 days post-fertilization they observed significant craniofacial defects with severe hypoplasia of the viscerocranium, including complete loss of branchial arches 3–7 and Meckel and ceratohyal cartilage (Van Laarhoven et al., 2015). When these structures were present, they were often incompletely formed or clefted. In addition, at 48 h post-fertilization Kmt2d morphants exhibited abnormal development of the atria and/or ventricle as well as prominent bulging of the myocardial wall, and progression through cardiac looping morphogenesis was significantly lower than that observed with wildtype. Compared to wildtype embryos, cross-sectional areas of morphant brains were notably reduced and had a reduced cell layer thickness within the hypothalamus, optic tectum, and midbrain tegmentum. Analysis of neural precursor cell (NPC) markers demonstrated that morphant NPCs are defective in their ability to differentiate in the forebrain and midbrain; the differentiation defects were not observed in the hindbrain.

##### Craniofacial features

The craniofacial characteristics of the KS include microcephaly, short columella, flat broadened tip of the nose, arched eyebrows, long eyelashes, long palpebral fissures with eversion of lateral parts of the lower lids, and large protruding or cupped earlobes (Wessels et al., 2002; Spano et al., 2008; Teixeira et al., 2009; Tuna et al., 2012).

Cleft palate is seen in almost half of the KS patients while a high arched palate is also a common finding (Adam and Hudgins, 2005; Schrander-Stumpel et al., 2005). Malocclusions, such as open bites, are commonly observed (Matsune et al., 2001; Tuna et al., 2012), as also are unilateral posterior cross bite (Matsune et al., 2001) and Angle class III malocclusion (do Prado Sobral et al., 2013).

##### Oral and dental features

The majority of the patients show hypodontia, mainly with agenesis of incisors and /or premolars (Teixeira et al., 2009). Other manifestations include microdontia and “screwdriver”—or peg-shaped incisors (Matsune et al., 2001; Adam and Hudgins, 2005; Rocha et al., 2008). Also supernumerary teeth and widely spaced teeth have been reported (Kuroki et al., 1981; Matsune et al., 2001; Petzold et al., 2003; Rocha et al., 2008). Retention of primary (Rocha et al., 2008) and permanent teeth (Petzold et al., 2003), as well as ectopic upper molars (Cogulu et al., 2008) are commonly observed disturbances of tooth eruption.

#### Kallmann syndrome

##### General features and genetics

Congenital hypogonadotropic hypogonadism (CHH) is a genetically heterogeneous syndrome caused primarily by gonadotropin-releasing hormone deficiency. About half of the CHH patients suffer from a reduced or deficient sense of smell (hyposmia or anosmia). This subtype of CHH is called Kallmann syndrome (KALS) (Boehm et al., 2015; Yoon et al., 2015).

Most Kallmann cases are diagnosed during infancy in males with cryptorchidism, micropenis, or associated non-reproductive signs or at the time of puberty due to lack of sexual development in combination with hyposmia or anosmia in both sexes. Different forms of KALS have been described, based on their genetic background. Kallmann−1 (KAL-1) is caused by X-linked recessive mutations in *KAL1* on chromosome Xp22.3, sometimes in association with a mutation in another gene, e.g., *PROKR2*. A main feature of this type are mirror movements of the upper limbs (MacColl and Quinton, 2005; Dode and Hardelin, 2009). Kallmann-2 (KAL-2) and is caused by AD mutations in *FGFR1* on chromosome 8p11, sometimes in association with mutation in other genes, e.g., *FGF8* and *GNRHR*). Approximately 30% of the KAL-2 type 2 cases are caused by *de novo* mutations (Sato et al., 2004; Zenaty et al., 2006; Dode and Hardelin, 2009; Boehm et al., 2015).

##### Craniofacial features

Cleft palate is reported in approximately 13% of the patients with KAL-1 (Honig, 1992; Molsted et al., 1997; Zenaty et al., 2006). Kallmann patients show an increased angulation of the mandible accompanied by an extreme mandibular and maxillary retrognathia (Molsted et al., 1997).

##### Oral and dental features

Tooth agenesis occurs in about 50% of KAL-1 syndrome (Honig, 1992; Molsted et al., 1997; Zenaty et al., 2006) both in cleft and non-cleft patients. It is highly variable, ranging from one to multiple congenitally missing teeth. The most frequently missing teeth are the lateral mandibular incisors, second mandibular and maxillary premolars, and lateral maxillary incisors. Apart from tooth agenesis microdontia, typical “screwdriver”-shaped mandibular incisors, and thin molar roots have also been observed in these patients (Molsted et al., 1997; Sato et al., 2004; Dode and Hardelin, 2009; Bailleul-Forestier et al., 2010).

#### Pierre Robin sequence

##### Genetics and general features

Pierre Robin syndrome is first described by the French surgeon Pierre Robin (Robin, 1923, 1934). However, it is nowadays called Pierre Robin Sequence (PRS), since its multiple anomalies result from a sequential chain of malformations, one entailing the next (Butow et al., 2009; Gangopadhyay et al., 2012). The primary cause is probably a growth defect of the embryonic mandible due to mutation in the *SOX9* gene. Activity of SOX9 is of importance for the formation of Meckel's cartilage and other chondral structures in the skull. Also, cartilaginous structures derived from the second and third branchial arches, such as stapes, the hyoid, the styloid, and the thyroid, are affected by the *SOX9* mutation (Mori-Akiyama et al., 2003). In a study of 2,530 non-syndromic cleft trios, Ghassibe-Sabbagh et al. (2011) found the *FAF1* locus to be strongly associated with cleft palate (Ghassibe-Sabbagh et al., 2011). In about half of the cases, PRS is associated with another syndrome, such as 22q11.2 deletion, Stickler, van der Woude, Möbius, and many others (Yu et al., 2005; Butow et al., 2009; Gangopadhyay et al., 2012; Ansari et al., 2014). Looking for the causal genetic variant in a 4-generation family with PRS, Benko et al. (2009) sequenced 4 candidate genes *SOX9, KCNJ2, KCNJ16*, and *MAP2K6*, but did not find any genomic changes or coding-sequence mutations (Benko et al., 2009). Performing an array comparative genomic hybridization (aCGH) analysis, a heterozygous 75-kb deletion located 1.38-Mb centromeric to the *SOX9* gene was identified. This region also encompassed 10 highly conserved noncoding elements (HCNEs) that segregated with the PRS. In 11 additional unrelated patients with PRS the aCGH analysis revealed *de novo* deletions in 2 sporadic cases, also involving a centromeric deletion (of more than 319 kb) and a telomeric 36-kb deletion, respectively. DNA sequencing of the 10 HCNEs in the remaining nine individuals with PRS revealed a heterozygous T-C transition in 1 family within an HCNE with features of a developmental enhancer *in vitro* and *in vivo*. The *in vitro* enhancer function was abrogated by the mutation and altered the binding of the MSX1 transcription factor compared to wildtype. In the developing mouse mandible, the 3-Mb region bounded by the microdeletions showed a regionally specific chromatin decompaction in cells expressing *SOX9*. Thus, PRS may also result from developmental misexpression of *SOX9* due to disruption of long range cis-regulatory elements (Benko et al., 2009).

##### Craniofacial features

In 75–100% of the cases with PRS, a cleft palate is a common finding (Butow et al., 2009; van Lieshout et al., 2014; Cote et al., 2015). PRS patients have a variable mandibular morphology and position depending on the occurrence and type of the associated syndromes (Rogers et al., 2009). However, if patients with isolated PRS are compared with unaffected control individuals, the mandibular length is significantly smaller and the ratio between ramus height and mandibular body is higher as also is the gonial angle (Suri et al., 2010; Boyce et al., 2012). Furthermore, non-syndromic PRS patients show a smaller cranial base and maxillary length, and increased palatal and mandibular plane inclinations (Suri et al., 2010). There is no evidence of the mandible showing an adolescent catch-up growth (Daskalogiannakis et al., 2001; Suri et al., 2010), which will lead to mandibular micrognathia (Mori-Akiyama et al., 2003; Yu et al., 2005; Cote et al., 2015).

##### Oral and dental features

The tongue develops in an abnormal dorsal position (glossoptosis), which can result in glossopharyngeal-laryngeal respiratory obstruction, vagal syncope, and feeding problems (Gangopadhyay et al., 2012).

The prevalence of tooth agenesis in the permanent dentition of PRS patients, is reported to range between 30 and 50% (excluding the 3rd molars), with the prevalence in the mandible being higher than in the maxilla. The most affected teeth are mandibular 2nd premolars (Ranta, 1986; Andersson et al., 2010, 2015; Antonarakis and Suri, 2014).

#### Van der Woude syndrome

##### Genetics and general features

Van der Woude syndrome (VWS) is the most frequent form of syndromic clefting (Rintala and Ranta, 1981) and accounts for 2% of all CLP patients. It was first described in 1954 (Van Der Woude, 1954) and is characterized by paramedian lip pits and sinuses, the second cardinal sign being orofacial clefting. VWS is genetically heterogeneous with VWS1 (OMIM #119300) being caused by heterozygous mutations in *IRF6* on chromosome 1q32.2 and VWS2 (OMIM #606713) by heterozygous mutations in *GRHL3* on chromosome 1p36.11 (Peyrard-Janvid et al., 2014). While epistatic genetic interaction was previously demonstrated between *p63* and *IRF6* (Thomason et al., 2010), this could not be demonstrated between *Irf6* and *Grhl3* (Peyrard-Janvid et al., 2014). *GRHL3* however belongs to a group of epidermal genes, which are significantly downregulated by mutations in AEC-related *TP63* mutations (Zarnegar et al., 2012). Recently mutations in *IRF6* were also shown to cause non-syndromic OFCs (Leslie et al., 2016; Khandelwal et al., 2017).

Like other individuals with OFC, especially cleft palate, patients with VWS have delayed language development, and mild cognitive problems (Nopoulos et al., 2002, 2007a,b).

Nopoulos et al. (2002) reported anterior cerebrum changes in brain MRI, in 14 adults with VWS. The intelligence score was lower and men were more affected than women (Nopoulos et al., 2007b). Other associated features of the syndrome include congenital heart disease, limb abnormalities (syndactyly of the hands, thumb hypoplasia, club foot), and sensorineural hearing loss (Rizos and Spyropoulos, 2004).

##### Craniofacial features

Clefts are reported in 21–100% of the VWS patients (Janku et al., 1980; Onofre et al., 1997) with different degree of severity (Onofre et al., 1997; Rizos and Spyropoulos, 2004).

VWS patients show an underdevelopment of the maxillary sagittal length and maxillary height. This is also reflected in a reduced ANB angle (Kane et al., 2002; Oberoi and Vargervik, 2005a). A more recent study (Heliovaara et al., 2015), did not support these findings. Compared to non-syndromic OFC patients a smaller diameter of the lower pharyngeal airway was observed in VWS patients (Heliovaara et al., 2015).

##### Oral and dental features

At birth, approximately 88% of the patients with VWS present with: paramedian lower-lip pits (usually bilateral) and/or a sinus tract leading from a mucous gland of the lip. These pits may show continuous or intermittent dribbling of watery or salivary secretions (Janku et al., 1980; Rintala and Ranta, 1981; Rizos and Spyropoulos, 2004). Hypodontia and dental hypoplasia are also considered cardinal features in VWS patients (Rizos and Spyropoulos, 2004; Hoefele et al., 2013). The prevalence is related to the severity of the cleft. Unilateral and bilateral cleft lip and palate (UCLP and BCLP) patients all have missing teeth, while in the isolated cleft palate and in the submucous cleft palate patients, missing teeth are seen in about 50% of the cases (Oberoi and Vargervik, 2005a). When all VWS patients are considered as a whole, tooth agenesis is reported in up to 80% of the cases (Ranta and Rintala, 1982; Rizos and Spyropoulos, 2004; Lam et al., 2010). The most affected teeth are the maxillary second premolars, followed by the maxillary lateral incisors (Ranta and Rintala, 1982). Conical elevations of the lower lip (Hoefele et al., 2013) and ankyloglossia (Rizos and Spyropoulos, 2004) are also among the findings of VWS. In order to gain insight into the molecular mechanisms contributing to the frequent co-occurrence of syndromic or non-syndromic tooth agenesis and OFCs, a systematic review revealed 84 articles including phenotype and genotype description of 9 genomic loci and 26 gene candidates underlying the co-occurrence of the combined congenital defects, one of them being *IRF6* (Phan et al., 2016). In another study the authors hypothesized that the expression of CLP genes may persist in the dental epithelium and thus, in addition to their role in labiopalatine development, may play an important functional role in subsequent tooth patterning and amelogenesis. Using a novel dental epithelium-specific *Irf6* conditional knockout (*Irf6*-cKO) mouse, Chu et al. (2016) were able to assess all post-eruption dental phenotypes, which include variable hypodontia, occasional supernumerary incisors and molars, as well as, crown and root patterning anomalies, peg-shaped first molars and taurodontic and C-shaped mandibular second molars (Chu et al., 2016). In addition, enamel density was reduced compared to control mice, ameloblasts exhibited disturbances in adhesion and polarity, and delayed enamel formation was observed. As these findings could perfectly be explained by the role of Irf6 in the organization of polarity of epithelial cell types, this data reinforced the notion that the various isolated tooth defects could be considered as part of the CLP spectrum in relatives of an affected individual (Chu et al., 2016). This also could explain earlier observations on a significantly higher frequency of solitary tooth agenesis in sibs of patients with non-syndromic OFCs (Eerens et al., 2001). On the other hand no significant *IRF6* variants could be identified in 67 patients with solitary (familial) tooth agenesis, but without sibs affected with OFCs (Khandelwal et al., 2017). This could however be due to the relatively small patient cohort.

### Syndromes with unusual faces

The syndromes with unusual faces described as well as their synonyms are presented in Table S1.

#### Coffin-Lowry syndrome

##### Genetics and general features

Coffin-Lowry syndrome (CLS) is caused by mutation in the *RSK2* gene (*RPS6KA3*) on chromosome Xp22.12. Its incidence is estimated 1 in 50,000–100,000 (Pereira et al., 2010). While inheritance occurs in an X-linked dominant manner, 70–80% of the patients are sporadic (Pereira et al., 2010). CLS was described by Coffin et al. (1966) and later by Lowry et al. (1971) as a different entity. Temtamy et al. (1975) showed that the two entities represented the same syndrome and since then is called CLS. Besides cognitive impairment, this rare X-linked intellectual disability syndrome, is also characterized by mainly skeletal malformations, osteopenia, and growth retardation. The causal gene, *RSK2*, was identified in 1996 and contains 22 exons which encode a protein of 740 amino acids (Hanauer and Young, 2002). Mutations in the ribosomal S6 kinase RSK2 encoding a growth factor-regulated kinase are required for osteoblast differentiation and function, were identified to cause the skeletal phenotype and osteopenia in individuals with CLS. Moreover, lack of the transcription factor ATF4, which was identified as a critical substrate for phosphorylation by RSK2, was found to dysregulate osteoblast differentiation and function like in patients with CLS (Yang et al., 2004). Women are more severely affected than men, and the disorder had a wide range of severity. Affected newborn males often show joint hyperlaxity and hypotonia. The hands are broad with soft, stubby, tapering fingers which may also be present at birth and are a strong diagnostic feature. Physical growth and psychomotor development is delayed and it is obvious since the very early age. Other typical general symptoms are short stature (95%), pectus deformity (80%), hearing deficit (sensorineural hearing loss), paroxysmal movement disorders and kyphosis and/or scoliosis which are seen in ~80% of the male individuals with CLS (Touraine et al., 2002). Cardiomyopathy including mitral valve dysfunction, has also been reported (Facher et al., 2004). In a brain morphometric study individuals with CLS consistently showed markedly reduced total brain volume. Particularly the cerebellum and the hippocampus were affected in patients diagnosed with CLS compared to controls (Kesler et al., 2007).

##### Craniofacial features

Craniofacial abnormalities in babies with CLS are not specific and it is only by the 2nd year of life that the typical facial characteristics of the syndrome become apparent. Most affected boys and some affected girls show typical craniofacial features like prominent forehead, hypertelorism, flat nasal bridge, downward sloping of palpebral fissures, large and prominent ears, and a wide mouth with full lips. All these characteristics progress mildly as the patient grows (Touraine et al., 2002; Lopez-Jimenez and Gimenez-Prats, 2003). Malocclusions, including frontal open bites (unpublished observations 2014, by author C.C.), and a high narrow palate are often present (Gilgenkrantz et al., 1988).

##### Oral and dental features

Hypodontia is seen in 85% of the males. Peg shaped or absent upper lateral incisors are also common (Pereira et al., 2010). Large divergent central incisors, with a wide upper interincisal diastema are also observed in CLS (Gilgenkrantz et al., 1988; Lopez-Jimenez and Gimenez-Prats, 2003), as also is a lingual midline fissure (Gilgenkrantz et al., 1988). As alveolar bone loss and premature tooth exfoliation were also consistently reported symptoms in patients with CLS, a Rsk2-deficient mouse model was generated, and the results showed that besides the requirement for alveolar bone formation, Rsk2 is also a critical regulator of cementoblast function. Using multiple techniques, it was demonstrated that Rsk2 is necessary for proper acellular cementum formation. Besides cementum hypoplasia, Rsk2 deficiency also causes detachment and disorganization of the periodontal ligament and is associated with significant alveolar bone loss with age. Rsk2 was thus identified as a non-redundant regulator of cementum homeostasis, alveolar bone maintenance, and periodontal health, with all these features being independent of Rsk2 function in systemic bone formation (Koehne et al., 2016).

#### Opitz GBBB syndrome

##### Genetics and general features

The Opitz GBBB syndrome is genetically heterogeneous of which an X-linked recessive and an autosomal dominant form have been described, with their signs and symptoms being in general the same. The X-linked form of Opitz GBBB syndrome-type1 (Opitz-GBBB1) is caused by mutation in the *MID1* gene on Xp22.2 and transmitted in an X-linked recessive way. It is characterized by several abnormalities along the midline of the body. Mutations in *MID1*, which encodes a microtubule-binding protein, have been found in ~50% of Opitz GBBB syndrome patients consistent with the genetically heterogeneous nature of the disorder (Short et al., 2002). The Opitz GBBB syndrome-type2 (Opitz-GBBB2) is caused by *SPECC1L* gene located on 22q11,23 (Kruszka et al., 2015).

Common general features for GBBB1 and GBBB2 are laryngo-tracheo-oesophagal abnormalities, and urogenital defects like hypospadias, cryptorchidism, and bifid scrotum in males and splayed labia majora in females, imperforate anus, and congenital heart defects. Mild intellectual disability and developmental delay occur in about 50% of the individuals with Opitz GBBB1-2 syndrome, and some patients present features of autistic spectrum disorders (Wilson and Oliver, 1988; Meroni, 1993; Quaderi et al., 1997; Jacobson et al., 1998; De Falco et al., 2003; Parashar et al., 2005; Aranda-Orgilles et al., 2008). Five patients were previously described with the Opitz (GBBB) syndrome phenotype and 22q11.2 deletion determined by fluorescent *in situ* hybridisation (FISH) but the precise limits of their deletions have not been determined. Since one locus of Opitz syndrome maps to 22q11.2 chromosomal arrangements are frequently complex and can inactivate such a locus. Erickson et al. (2007) performed high-resolution array-based comparative genomic hybridization (CGH) on a new Opitz syndrome-like phenotype patient with a 22q11.2 deletion. This patient shares the same deletion as patients with velocardiofacial (VCF) and DiGeorge syndrome (see earlier in this review).

##### Craniofacial features

Distinct craniofacial features that may be seen in this disorder include a prominent forehead, with a widow's peak hairline, hypertelorism, a flat nasal bridge, a thin upper lip, and low-set ears (Hsieh et al., 2008). Cleft lip and/or palate is seen in ~50% of the individuals (Meroni, 1993; Parashar et al., 2005; Aranda-Orgilles et al., 2008; Hsieh et al., 2008; Bhoj et al., 2015).

##### Oral and dental manifestations

Only one case history is published that describes neonatal mandibular incisors in two brothers from one family (Shaw et al., 2006).

#### Smith-Lemli-Opitz syndrome

##### Genetics and general features

Smith-Lemli-Opitz syndrome (SLOS) is a developmental disorder that affects many parts of the body, and is caused by a homozygous or compound heterozygous mutation in the gene encoding sterol delta-7-reductase (*DHCR7*) located on chromosome 11q13, with autosomal recessive inheritance (Tint et al., 1995). The syndrome has been initially described by Smith et al. (1964). DHCR7 is an enzyme for the biosynthesis of cholesterol of which over 160 different mutations have been described, and the severity of the physical effects of the mutations correlates with the severity of the cholesterol deficiency (Tint et al., 1995; Tanwar et al., 2013) leading to the syndrome with a variable phenotype severity (Saher and Stumpf, 2015). SLOS therefore constitutes a clinical and biochemical continuum. The discovery of the deficiency of DHCR7 as a cause for the SLOS (Tint et al., 1995) made this syndrome the first true metabolic syndrome of multiple congenital malformations. About 80% of the patients show limb deformities such as syndactyly or polydactyly. Almost all patients show learning disabilities and mental deficiencies. Congenital heart deformities, gastrointestinal problems and urogenital anomalies have also been described (Antoniades et al., 1994; Muzzin and Harper, 2003; Pizzo et al., 2008).

The wide variability of the signs and symptoms of SLOS, may range from mildly affected individuals with minor learning and behavioral abnormalities and physical deviations till severe life-threatening forms of the syndrome.

Quintana et al. (2017) reported on eight different human syndromes caused by mutations in the cholesterol synthesis pathway (Quintana et al., 2017). A subset of these disorders such as SLOS, is associated with facial dysmorphia. However, the molecular and cellular mechanisms underlying such facial deficits are not fully understood, primarily because of the diverse functions associated with the cholesterol synthesis pathway. Their data raise a novel function for isoprenoids in facial development and collectively suggest that cholesterol regulates craniofacial development through versatile mechanisms (Quintana et al., 2017). These authors conclude that future studies deciphering the downstream pathways modulated by cholesterol and isoprenoids will be necessary to gain a more comprehensive understanding of neural crest associated human diseases, including SLOS.

##### Craniofacial features

Craniofacial characteristics of this syndrome include microcephaly, bitemporal narrowing, ptosis, short nose with anteverted nares, low-set and retroversed ears, ocular problems and hypertelorism, a small chin, and micrognathia (Antoniades et al., 1994; Muzzin and Harper, 2003; Pizzo et al., 2008).

Other clinical features include cleft palate or bifid uvula that is related to the increased level of 7DHC (Saher and Stumpf, 2015). Cleft palate has been reported in 40–50% of the patients (Cunniff et al., 1997; Muzzin and Harper, 2003; Porter, 2006). Cleft lip is however uncommon (Rajpopat et al., 2011).

##### Oral and dental features

The oral and dental manifestations in SLOS individuals include, oligodontia or supernumerary teeth, broad alveolar ridges, enamel hypoplasia, protrusion of the maxillary front teeth, lip incompetence and an anterior open bite (Antoniades et al., 1994; Pizzo et al., 2008).

## Discussion

In this review we updated recent advances in genetic etiology and genotype-phenotype relations of 13 syndromes affecting craniofacial and dental structures. Considerable phenotype variability is reported in literature, also among the affected individuals of one family with the same causal variant. For better understanding the pathogenic mechanisms of these syndromes, the disrupted molecular pathways as well the prioritized HPO terms (Köhler et al., 2017), were presented in Table 1A. A frequency count in this table suggests for example that in 7 different craniofacial syndromes, i.e., NF1, HFM, TCS1, Kallmann syndrome, Pierre Robin sequence, Kabuki Syndrome and VCFS, enrichment was found for abnormalities of the cardiovascular system (HP:0001626) including abnormalities of cardiac septa (HP:0001671) and of the aortic arch (HP:0012303). Importantly, the HPO term, abnormalities of the cardiovascular system (HP:0001626) thereby emerges as the most frequent common phenotype of the selected syndromes. Interestingly, the HPO term “abnormalities of bone mineral density” (HP:0004348) was exclusively enriched in the three TCS-forms, while “abnormalities of the cranial nerves” (HP:0001291), was unique for Hemifacial Microsomia syndrome. On the other hand, the HPO term for “abnormalities of dental enamel” (HP:0000682) was enriched in 5 of our entities, namely in TCS1, TCS2, TCS3, VCFS, and EEC3 syndrome. In TCS1, TCS2, TCS3, and VCSF, enrichment was also found in “abnormalities of dental morphology” (HP:0006482). The latter term also overlapped with “abnormalities of dental enamel” (HP:0000682), and with CLS as an additional condition (Table 1A).

When the GO terms for preferred cellular component, molecular function, and biological processes (Ashburner et al., 2000; The Gene Ontology Consortium, 2015) were analyzed in Table 1B, the causal mutant proteins were enriched in the nucleus or in the nuclear membrane. On the other hand their most frequently involved molecular functions appear to be “protein binding” (GO:0005515) including “chromatin binding” (GO:0003682) and “DNA binding, including regulatory region DNA binding” (GO:0000975) and “transcription regulatory region sequence specific binding” (GO:0000976). The GO terms for the biological process involvement were more diverse. The highest enriched functions were regulatory activities, skeletal system development (GO:0001501) and angiogenesis (GO:0001525 and GO:0001568).

Combining deep clinical phenotyping, eventually aided with 3D facial imaging (Vink et al., 2014; Lewyllie et al., 2017) and including the use of HPO and GO terms as above, we could promote interdisciplinary interaction and contribute to a mechanistic diagnosis. Moreover, pathway information (Table 1A) could also provide clues for molecular preventive counseling in parents at risk, in the future. In this respect, the experiments in mouse of Sakai et al. (2016) can be considered as a breakthrough. While Dixon et al. (2007) showed that Tcof1 haploinsufficiency results in oxidative stress-induced DNA damage with neuroepithelial cell death. Sakai et al. (2016) demonstrate that maternal treatment with antioxidants minimizes cell death in the neuroepithelium and substantially ameliorates or even prevents the pathogenesis of craniofacial anomalies in Tcof1(+/−) mice. The authors conclude that antioxidant therapy may provide an avenue of protection against the pathogenesis of TCS and similar neurocristopathies (Sakai et al., 2016).

Finally, we would like to address some practical issues for a better mutual understanding of the specialists in the interdisciplinary guiding team. First of all “deep phenotyping” will be postponed in syndromes with involvement of the dentition. While the deciduous dentition is normally completed around the age of 3, the permanent dentition (with exception of the wisdom teeth) will be completely erupted by around 14 years of age. This definitely postpones the full phenotyping in patients diagnosed with one of the syndromes affecting craniofacial, oral, and dental structures.

Secondly, all team members should be aware of the fact that treatment of facial mimic together with other facial morphologic corrections (Ferrari et al., 2017; Magnifico et al., 2017), post-operative physiotherapy (Gaudin et al., 2016; McKay et al., 2016) and orthodontic treatment can improve function and esthetics in patients diagnosed with conditions described in our review (Ferrari et al., 2017; Magnifico et al., 2017).

In growing individuals with craniofacial asymmetries like patients with NF1 or HM, the use of hybrid functional orthodontic appliance (Nouri and Farzan, 2015) or distraction osteogenesis (DO) (Chauhan and Guruprasad, 2015) is the treatment of choice in mild to moderate asymmetries. Bimaxillary DO has been suggested as treatment alternative in non-growing patients (Sant'Anna et al., 2015; Liu et al., 2017). Since DO has not given always satisfactory results in severe craniofacial asymmetries (Lu et al., 2016; Luo et al., 2016) the staged orthognathic surgery is advocated (Liu et al., 2017). Stability of the treatment outcome is an issue in the facial asymmetries and craniofacial discrepancies. Overcorrection of orthognathic treatment and surgery planning only at the end of craniofacial growth, individualized according to the patient and the syndrome, can limit esthetic complications and additional surgical procedures (Fattah et al., 2014).

Focusing on the etiopathogenesis like the facial nerve palsy in HM patients and not only in skeletal and soft tissue deficiencies results in better esthetic and functional treatment outcome (Choi et al., 2014, 2015).

Many of these patients have reduced nasal airway volume like the TCS patients (Ma et al., 2015) resulting in impaired phonation and OSA (Plomp et al., 2015). ENT detailed examination is required for identification of important components for future treatment interventions (Plomp et al., 2015). In many cases the orthognathic correction, like the mandibular DO increases the airway and prevents the OSAS (Damlar et al., 2016). Articulatory dysfunction should be improved or corrected for proper social integration and quality of life of these children (Golinko et al., 2016).

In conclusion, the early detection of syndromes affecting craniofacial and dental structures, improves genetic diagnostic counseling and long-term treatment planning (Dentici et al., 2015). However, as dental development and facial growth are lagging behind (being normally only complete around 14 and 18 years of age, respectively), deep phenotyping of craniofacial syndromes will only be possible in young adulthood. This makes early interventions and decisions concerning the type and timing of orthodontic treatment and maxillofacial surgery often difficult and critical, especially in patients with disrupted development in the craniofacial and dental structures.

## Author contributions

TB and JM: conceived the idea; TB, CC, and JM: performed the literature search. TB and CC: wrote the review and finalized the work; JM: edited the text and tables.

### Conflict of interest statement

The authors declare that the research was conducted in the absence of any commercial or financial relationships that could be construed as a potential conflict of interest.
