# Supplementary material for: Update on 13 Syndromes Affecting Craniofacial and Dental Structures
Source: Front Physiol. 2017 Dec 14;8:1038. doi: 10.3389/fphys.2017.01038 (PMC5735950; doi:10.3389/fphys.2017.01038)
Supplement: Supplementary file 1 [file Table1.docx]

| **Syndromes involving gingivodental tissues** | **Synonyms** | **OMIM / Orpha** | **Prevalence**  **per 100,000** | **General features** | **Craniofacial features** | **Oral and dental features** |
| --- | --- | --- | --- | --- | --- | --- |
| Neurofibromatosis  type 1 | Von Recklinghausen disease Peripheral Neurofibromatosis | 162200 /  636 | 25.0-33.3 | - Neurofibroma, cancerous tumors, (Garcia-Romero, 2015) - café-au-lait spots, iris Lisch nodule (Kehrer-Sawatzki et al., 2017) - skeletal abnormalities (scoliosis etc.), hypertension (Garcia-Romero et al., 2015; Ruggieri et el., 2001) | - Macrocephaly, short mandible, maxilla, cranial base, and low face height (Heervaara et al., 2011;Cung et al., 2015) - enlargement of the mandibular canal (Visnapuu et al., 2012) - Class III molar relationship, notching of the posterior border of the mandibular ramus, increased length of the coronoid process, hypoplastic condyles and zygomatic processes (Bardellini et al., 2011;Javed et al., 2014;Remberger et al., 1985;Scarano et al., 2005) - facial asymmetries and hypertelorisms (Kehrer-Sawatzki et al., 2017) | - Unilateral swelling of the gingiva (Javed et al., 2014) - melanin pigmentation of the gingiva (rare) (Javed et al., 2014) - impacted, supernumerary, displaced teeth or TA, periapical cementum dysplasia (Visnapuu et al., 2012;Friedrich et al., 2012) - tooth spacing (although size of tooth crowns is normal) (Friedrich et al., 2012) |
| **Syndromes involving branchial arches** |  |  |  |  |  |  |
| Hemifacial microsomia | Goldenhar syndrome  Oculo-auro-ventriculo-vertebral spectrum  Oculo-auro-ventriculo-vertebral dysplasia  Facio-auriculo-vertebral sequence | 164210 /  374 | 1.8 – 3.9 | - cardiac, neural, renal vertebral, ocular and central nervous system defects (Akram et al., 2015; Gorlin et al., 1963) | - external and middle ear deformity, hypoplasia of the zygomatic, mandibular and maxillary bones, facial muscle hypoplasia, coloboma’s of the upper eyelids, orbit, eye, nose, cranium, or neck, upward cant of the occlusal plane, small mandibular body and ramus (Heike et al., 2016;Brandstetter et al., 2016;Shibazaki-Yorozuya et al., 2014) - facial nerve palsy, mandibular condyle hypoplasia, soft tissue discrepancy, craniofacial growth asymmetry (Choi et al., 2014) - normal mandibular growth rate (despite asymmetry) (Ongkosuwito et al., 2013) - steep and retrognathic mandibles large gonial angles, mildly convex profile (Seow et al., 1998;Ongkosuwito et al., 2013 ;Ahiko et al., 2015), CLP (Ye et al., 2005) - OSA (Szpalski et al., 2015) | - hypoplastic molars (in both dentitions) (Seow et al., 1998) - TA (Ongkosuwito et al, 2010;Maruko et al., 2001) - delayed tooth development, absent mandibular ramus and glenoid fossa (severe cases) (Ongkosuwito et al., 2010;Ahiko et al., 2015) - tongue dysmorphisms (Chen et al., 2009) |

Table S1 Syndromes affecting craniofacial and dental structures: Synonyms, OMIM/Orpha, prevalence, general, craniofacial, oral and dental features.

| Treacher Collins 1 | Treacher-Collins syndrome  Treacher-Collins-Francetti syndrome  Mandibulafacial dysostosis | 154500 / 861 | 2.0 | - encephalocele, extracraniofacial anomalies of thyroid, thymus, heart, accessory spleen, ectopic adrenal gland tissue, underdeveloped external genitalia (Li et al., 2009) | - craniofacial and downward-slanting palpebral fissures, malar and mandibular hypoplasia (Vincent et al., 2016) - narrow arched palate, maxillary hypoplasia, hypoplasia of soft tissues of the face, (Posnick et al., 2000;Poswillo et al., 1975) - anterior open bite (Posnick et al., 2000; Martelli-Junior et al., 2009; Trainor et al., 2013;Posnick et al., 2000), - cleft palate (Vincent et al., 2016 ;Posnick et al., 2000; da Silva Dalben et al., 2006; Martelli-Junior et al., 2009) - TMJ abnormalities (Posnick et al., 2000 ; Poswillo et al., 1975) - atresia of external ear canal, microtia, coloboma of the lower eyelid, facial asymmetry and projection of scalp hair onto the lateral cheek, choanal stenosis or atresia (Vincent et al.,2016) - disturbed respiration, OSAS (Akre et al., 2012) | - Dental anomalies, TA (da Silva Dalben et al., 2006) - impacted supernumerary teeth (da Silva Dalben et al., 2006) - ectopic eruption of the maxillary first molars (da Silva Dalben et al., 2006) - open bite (Martelli-Junior et al., 2009) |
| --- | --- | --- | --- | --- | --- | --- |
| Treacher Collins 2 | / | 613717 /  861 |  |  |  |  |
| Treacher Collins 3 | Mandibulofacial dysostosis,  Treacher-Collins type *(autosomal recessive*) | 248390 / 861 |  |  |  |  |
| Möbius | Moebius syndrome  Moebius sequence  Congenital facial diplegia | 157900 /  570 | 0.2 – 2.0 | - absence of the 7^th^ cranial nerve (facial), and the 6^th^ cranial nerve, other cranial nerves can also be affected (Verzijl et al., 2003) - mild intellectual deficit (Verzijl et al., 2003) - behavioral, and cognitive abnormalities, limb, musculoskeletal abnormalities (Van Der Zwaag et al., 2002 #1644;Ghosh et al., 2017;Verzijl et al., 2003) - several orthopaedic problems (clubfoot, scoliosis, and upper extremity deviations (McClure et al., 2016) - congenital heart diseases, association with other syndromes (Budic et al., 2016;Gaudin et al., 2016;Sharma et al., 2015) | - impaired ability to suck, drooling, orofacial dysmorphism, and jaw abnormalities, (Verzijl et al., 2003;Van Der Zwaag et al., 2002) - micrognathia and microstomia (De Serpa Pinto et al., 2002;Magalhaes et al., 2006 ;Bianchi et al., 2013) - narrow maxilla and high arched, cleft palate (De Serpa Pinto et al., 2002 ;Magalhaes et al., 2006;Stromland et al., 2002) - facial asymmetry and torticollis (Ghosh et al., 2017) - lack of facial expression, absence of blinking, strabismus, lateral eye muscle weakness (Bianchi et al., 2010;Sjogreen et al., 2001;Sjogreen et al., 2011;Zuker et al., 2000) - feeding and respiratory difficulties (Verzijl et al., 2003) | - hypoplastic upper lip, open bite (De Serpa Pinto et al., 2002;Magalhaes et al., 2006) - TA (Stromland et al., 2002), incompetent lips, failure of mouth closure (Ghosh et al., 2017) - short or fissured and unusually shaped tongue (De Serpa Pinto et al., 2002) |
| **Syndromes involving**  **oral clefts** |  |  |  |  |  |  |
| 22q11.2 deletion | CATCH22  Cayler cardiofacial syndrome  Conotruncal anomaly face syndrome (CTAF)  DiGeorge sequence  DiGeorge Syndrome  Microdeletion 22q11.2  Monosomy 22q11  Sedlackova syndrome  Shprintzen syndrome  Takao syndrome  Velocardiofacial syndrome | 192430 / 188400 /  567 | 25 - 50 | - cardiac anomalies, dysgenesis of the thymus and parathyroid glands, hypocalcaemia, immune deficiencies, learning disabilities, - disturbances in cognitive and behavioral development {Borglum Jensen et al., 1983;Ryan et al., 1997;Wang et al., 1997;Gaspar et al., 1999;Pradel et al., 2003;Yang et al., 2005; Nugent et al., 2010; Toka, 2010;   Wu et al., 2013)   - low height and weight of patients (Ryan et al., 1997) | - long, asymmetric face, hypotonic muscles, and microcephaly (Toka et al., 2010) - short philtrum, thick and reflected lips (Fukui et al., 2000;Wang et al., 1997) - bulbous-tipped nose, malar flattening, squared nasal root, narrow alar base, thin alae nasi, short velum, deep cavum, malformed or short cranial base (Leveau-Geffroy et al., 2011;Wang et al., 2009) - large cranial base angle (Oberoi et al., 2005) - micrognathia (Wang et al., 1997), retrognathia (Gaspar, 1999 #1902;Wang, 2009 #1889;Gaspar, 1999 #1902}, - steep mandibular plane angle, increased anterior face height, retruded chin, retroclined lower incisors, increased interincisal angle (Oberoi et al., 2005;Oberoi et al., 2011) - skeletal class II malocclusions, retruded mandible, open bite (Oberoi et al., 2011; Lewyllie et al., 2017) - hypoplasia of the lower part of the face (Lewyllie et al., 2017) - CP or palatal anomalies, hypertelorism (Wu et al., 2013;Nugent et al., 2010;Toka et al., 2010) - craniospinal growth disorders, functional impairment (Leveau-Geffroy et al., 2011) - asymmetric development of the pharynx and larynx, velopharyngeal insufficiency (Leveau-Geffroy et al., 2011;Nugent et al., 2010) - alternations in palatal motion, speech difficulties, respiration (Pradel et al., 2003;Chegar et al., 2006;Kummer et al., 2007) | - TA (Heliovaara et al., 2011;Lewyllie et al., 2017) - solitary median maxillary or mandibular central incisor (Oberoi et al., 2005 ;Yang et al., 2005) - delayed eruption of permanent teeth, enamel opacities (Fukui et al., 2000) - hypoplastic enamel alterations (Fukui et al., 2000;da Silva Dalben et al., 2008) - hypomineralizations (Nordgarden et al., 2012) - impaired salivary flow (Toka et al., 2010) |
| EEC 1 | Ectrodactyly, ectodermal dysplasia and cleft lip/palate syndrome | 129900 /  1896 | <0.1 | - Ectrodactyly, ectodermal dysplasia (Roelfsema et al., 1996;Rinne et al., 2006) - skin hypopigmentation and dry skin, hyperkeratosis, or atrophy, fine and sparse hair and eyebrows, nail dystrophy, reduction or absence of sweat, sebaceous and salivary glands (Buss et al., 1995 ;Rinne et al., 2006) - lacrimal tract abnormalities, ophthalmological problems, urogenital abnormalities, mammary gland/nipple hypoplasia - hearing loss (Maas, 1996;Roelfsema et al., 1996; Rinne et al., 2006) - hypothalamo-pituitary dysfunction (Gershoni-Baruch et al., 1997; Gershoni-Baruch et al., 1997) - growth hormone-deficiency (Knudtzon et al., 1987) - growth retardation (Roelfsema et al., 1996) | - orofacial cleft {Buss et al., 1995; Roelfsema et al., 1996) - CL only (Leslie et al., 2017) - midfacial, zygomatic, maxillary, and mandibular hypoplasia, microcephaly, premaxillary protrusion (*not typical characteristics of the syndrome*) (Roelfsema et al., 1996) - orofacial clefts of the lip only (Leslie et al., 2017) | - hypodontia (King et al., 1994), or even anodontia (Wallis et al., 1988) - enamel hypoplasia (Knudtzon et al., 1987; King et al., 1994; Leibowitz et al., 1984; Wallis et al., 1988;Turner et al., 2005) - generalized microdontia (King et al., 1994) - poorly developed teeth (Wallis et al., 1988) - peg-shaped teeth (Leibowitz et al., 1984; Wallis et al., 1988) - oral candidiasis (King et al., 1994) - chronic ulcerative stomatitis (Romano et al., 2012) - xerostomia and /or deep tongue fissures (King et al., 1994) - late dental age and tooth eruption (Klein et al., 2013) |
| EEC 3 |  | 604292 /  2440 | 5.4 |  |  |  |
| Kabuki 1 | Kabuki makeup syndrome  Niikawa-Kuroki syndrome | \|  \| \| --- \|   147920 /  2322 | 3.2 (Japan) | - growth delay (Cheon et al., 2015;Petersen et al., 2010; Adam et al., 2005; Schrander-Stumpel et al., 2005) - early puberty (Schrander-Stumpel et al., 2005) - intellectual disability (Wessels et al., 2002; Schrander-Stumpel et al., 2005; Vaux et al., 2005) - skeletal abnormalities (scoliosis, brachydactyly V, brachymesophalangy, clinodactyly of the fifth fingers, hypermobility and dislocation of hip and knee), seizures, muscle hypotonia (Burke et al., 1995) - heart abnormalities (Yoon et al., 2015; Yuan et al., 2013) - otitis media, hearing loss (Tekin et al., 2006; Kawame et al., 1999) - ocular problems (nystagmus, strabismus, ptosis) (Turner, 2005 #3200) - severe hypoplasia of the viscerocranium, complete loss of branchial arches 3 to 7 and Meckel and ceratohyal cartilage (Van Laarhoven et al., 2015) | - CP, high arched palate (Schrander-Stumpel et al., 2005;Adam et al., 2005) - open bite (Matsune et al., 2001;Tuna et al., 2012) - unilateral posterior cross bite (Matsune et al., 2001) - Angle Class III malocclusion (do Prado Sobral et al., 2013) - microcephaly, short columella, flat broadened tip of the nose, arched eyebrows, long eyelashes, long palpebral fissures with eversion of lateral parts of the lower lids, large protruding or cupped earlobes (Teixeira et al., 2009;Wessels et al., 2002;Tuna et al., 2012;Wessels et al., 2002;Spano et al., 2008) | - TA (Teixeira et al., 2009) - supernumerary teeth, widely spaced (Rocha et al., 2008;Kuroki et al., 1981; Matsune et al., 2001; Petzold et al., 2003) - microdontia, “screwdriver” - or peg-shaped incisors (Adam et al., 2005;Rocha et al., 2008; Matsune et al., 2001) - retention of primary (Rocha et al., 2008) and permanent teeth (Petzold et al., 2003) - ectopic upper molars (Cogulu et al., 2008) |
| **Kabuki 2** |  | 300867 /  2322 |  |  |  |  |
| Kallmann 1 | Anosmic hypogonadism  Anosmic idiopathic hypogonadotropic hypogonadism  Congenital hypo-gonadotropic hypogonadism with anosmia  Dysplasia olfacto-genitalis of De Morsier  Familial hypogona-dism with anosmia  Hypogonadotropic hypogonadism and anosmia  Hypogonadotropic hypogonadism-anosmia syndrome  H[ypothalamic](http://en.wikipedia.org/wiki/Hypothalamus) [hypogonadism](http://en.wikipedia.org/wiki/Hypogonadism)  Olfacto-genital pathological  sequence | 308700 /  478 | 12.5 (♂)  2.5 (♀) | - cryptorchidism, micropenis, lack of sexual development, hyposmia or anosmia, mirror movements of the upper limbs (Dode et al., 2009; MacColl et al., 2005) | - CP (Zenaty et al., 2006;Honig et al., 1992; Molsted et al., 1997) - increased mandibular angle, mandibular and maxillary retrognathia (Molsted et al., 1997) | - TA (Zenaty et al., 2006; Honig et al., 1992; Molsted et al., 1997) - microdontia, typical “screwdriver”-shaped mandibular incisors, thin molar roots (Bailleul-Forestier et al., 2010; Molsted et al., 1997; Sato et al., 2004; Dode, 2009) |
| Kallmann 2 |  | 147950 /  478 | 1.2 - 10 |  |  |  |
| Pierre Robin Sequence | Glossoptosis, micrognathia, and cleft palate  Pierre Robin malformation  Pierre Robin malformation sequence  Pierre Robin syndrome  Pierre Robin anomalad  Robin sequence  Robin syndrome | 261800 /  718 | 7.1 – 11.8 | - stapes, hyoid, styloid, thyroid are affected (Mori-Akiyama et al., 2003) | - CP (van Lieshout et al., 2014;Butow et al., 2009;Cote et al., 2015) - small mandibular length, ratio between ramus height and mandibular length is larger as is the gonial angle (Boyce et al., 2012;Suri et al., 2010) - smaller cranial base length, shorter maxillary length, increased palatal and mandibular plane inclinations (Suri et al., 2010) - mandibular micrognathia (Mori-Akiyama et al., 2003;Yu et al., 2005; Cote et al., 2015) | - Glossoptosis, airway-obstruction, feeding problems, glossopharyngeal- laryngeal respiratory obstruction, vagal syncope (Gangopadhyay, 2012). - TA, (Ranta, 1986; Andersson, 2015; Andersson, 2010 ; Antonarakis, 2014) |
| Van der Woude 1 syndrome | Cleft lip and/or palate with mucous cysts of lower lip  Lip-pit syndrome | 119300 /  888 | 1 – 3 | - delayed language development, learning disabilities, mild cognitive problems (Nopoulos et al., 2002; Nopoulos et al., 2007a; Nopoulos et al., 2007b) - congenital heart disease, limb abnormalities (syndactyly of the hands, thumb hypoplasia, club foot), sensorineural hearing loss (Rizos et al., 2004) | - OC (Onofre et al., 1997;Janku et al., 1980;Rizos et al., 2004) - smaller diameter of lower pharyngeal airway (Heliovaara et al., 2015) | - paramedian lower-lip pits (fistulae, usually bilateral), with watery or salivary secretions (Janku et al., 1980;Rintala et al., 1981;Rizos et al., 2004) - conical elevations of the lower lip (Hoefele et al., 2013), ankyloglossia (Rizos et al., 2004) - TA and dental hypoplasia (Hoefele et al., 2013;Rizos et al., 2004) - supernumerary teeth, root anomalies, peg-shaped first molars and taurodontic,C-shaped mandibular second molars (Chu et al., 2016) |
| Van der Woude 2  syndrome |  | \|  \| \| --- \|   606713 / 888 | 1 – 3 |  |  |  |
| **Syndromes with unusual faces** |  |  |  |  |  |  |
| Coffin-Lowry syndrome | Mental retardation with osteocartilaginous abnormalities | 303600 /  192 | 2-2.5 | - skeletal malformations (e.g.: kyphosis, scoliosis), hyperlaxity of joints (Touraine et al., 2002;Hanauer et al., 2002), osteopenia (Hanauer et al., 2002) - growth (Hanauer et al., 2002;Touraine et al., 2002) - psychomotor retardation (Touraine et al., 2002) - hearing deficit (sensorineural hearing loss) (Touraine et al., 2002) - cardiomyopathy (Facher et al., 2004) - reduced brain volume (Kesler et al., 2007) | - typical facial appearance usually apparent by 2 years, prominent forehead, hypertelorism, a flat nasal bridge, downward sloping of palpebral fissures, large and prominent ears, wide mouth with full lips (Touraine et al., 2002;Lopez-Jimenez et al., 2003) | - TA, peg shaped upper lateral and incisors are also common (Pereira et al., 2010) - interincisal (Gilgenkrantz et al., 1988;Lopez-Jimenez et al., 2003) - lingual midline fissure (Gilgenkrantz et al.,1988) - alveolar bone loss, (Koehne et al., 2016) - malocclusions, including frontal open bites (unpublished observations 2014, by author C.C.), - high narrow palate (Gilgenkrantz et al., 1988) |
| Opitz G/BBB type 1 | Hypertelorism-oesophageal abnormality-hypospadias syndrome  Hypospadias-dysphagia syndrome  Hypospadias-hypertelorism syndrome  Opitz syndrome X-linked  Opitz-Frias syndrome | 300000 /  306597 | 2-10 | - abnormalities along the midline of the body (Short et al., 2002) - hypertelorism, CLP and/or uvula, laryngo-tracheo-oesophagal abnormalities, genito-urinary defects (e.g.: hypospadias, cryptorchidism , bifid scrotum in males and splayed labia majora in females), imperforate anus, congenital heart defects, mild intellectual disability, developmental delay features of autistic spectrum disorders (Aranda-Orgilles et al., 2008;Jacobson, 1998;Wilson et al., 1988;Parashar et al., 2005;De Falco et al., 2003;Meroni et al., 1993 ;Quaderi et al., 1997) | - prominent forehead, widow’s peak hairline, flat nasal bridge, thin upper lip, low-set ears (Hsieh et al., 2008) - CLP (Aranda-Orgilles et al., 2008; Parashar et al., 2005;Hsieh et al., 2008; Meroni et al., 1993;Bhoj et al., 2015) | - neonatal mandibular incisors in two brothers from the same family (only one case history is published) (Shaw et al., 2006) |
| Opitz G/BBB type 2 |  | 145410 /  306588 |  |  |  |  |
| Smith-Lemli-Opitz | 7-dehydrocholesterol reductase deficiency  RSH syndrome  SLOS | 270400 /  818 | 2.5-5 (Center/  Northern Europe) | - limb deformities (e.g.: syndactily or polydactyly), - learning disabilities, mental deficiencies, congenital heart deformities, gastrointestinal problems, urogenital anomalies (Muzzin et al., 2003; Pizzo et al., 2008 ; Antoniades et al., 1994) | - CP or bifid uvula (Saher et al., 2015; Cunniff et al., 1997;Muzzin et al., 2003; Porter et al.,, 2006) - CL is uncommon (Rajpopat et al., 2011) - microcephaly, bitemporal narrowing, ptosis, broad nasal bridge, short nasal root, anteverted nares, low-set and retroversed ears, ocular problems, hypertelorism, small chin, micrognathia (Antoniades et al., 1994;Pizzo et al., 2008; Muzzin et al., 2003) | - broad alveolar ridges, enamel hypoplasia, oligodontia or hyperdontia, protrusion of the maxillary front teeth, lip incompetence, open bite (Antoniades et al., 1994; Pizzo et al., 2008) |

CL, cleft lip; CLP, cleft lip and palate; CP, cleft palate; OC, Orofacial clefts; OMIM, Online Mendelian Inheritance in Man; Orpha, Orpha number; OSA, obstructive sleep apnea; OSAS, obstructive sleep apnea syndrome; TA, tooth agenesis; TMJ, Temporomandibular joint.
